# Mesenchymal Stem/Progenitor Cells: The Prospect of Human Clinical Translation

**DOI:** 10.1155/2020/8837654

**Published:** 2020-08-11

**Authors:** Dina Rady, Marwa M. S. Abbass, Aiah A. El-Rashidy, Sara El Moshy, Israa Ahmed Radwan, Christof E. Dörfer, Karim M. Fawzy El-Sayed

**Affiliations:** ^1^Oral Biology Department, Faculty of Dentistry, Cairo University, Cairo, Egypt; ^2^Stem Cells and Tissue Engineering Research Group, Faculty of Dentistry, Cairo University, Cairo, Egypt; ^3^Biomaterials Department, Faculty of Dentistry, Cairo University, Cairo, Egypt; ^4^Clinic for Conservative Dentistry and Periodontology, School of Dental Medicine, Christian Albrechts University, Kiel, Germany; ^5^Oral Medicine and Periodontology Department, Faculty of Dentistry, Cairo University, Cairo, Egypt

## Abstract

Mesenchymal stem/progenitor cells (MSCs) are key players in regenerative medicine, relying principally on their differentiation/regeneration potential, immunomodulatory properties, paracrine effects, and potent homing ability with minimal if any ethical concerns. Even though multiple preclinical and clinical studies have demonstrated remarkable properties for MSCs, the clinical applicability of MSC-based therapies is still questionable. Several challenges exist that critically hinder a successful clinical translation of MSC-based therapies, including but not limited to heterogeneity of their populations, variability in their quality and quantity, donor-related factors, discrepancies in protocols for isolation, in vitro expansion and premodification, and variability in methods of cell delivery, dosing, and cell homing. Alterations of MSC viability, proliferation, properties, and/or function are also affected by various drugs and chemicals. Moreover, significant safety concerns exist due to possible teratogenic/neoplastic potential and transmission of infectious diseases. Through the current review, we aim to highlight the major challenges facing MSCs' human clinical translation and shed light on the undergoing strategies to overcome them.

## 1. Introduction

Tissue engineering combines stem/progenitor cells with proper signaling molecules to be seeded on biocompatible scaffolds in the presence of physical stimuli to function in place of or to support regeneration of specific tissues or organs [1–3]. Mesenchymal stem/progenitor cells (MSCs) are key players in regenerative medicine, owing to their remarkable differentiation and regeneration potentials in addition to their immunomodulatory properties, paracrine effect [4, 5], and potent homing ability with no ethical concerns [6–8]. MSCs are multipotent cells, hallmarked by their ability to differentiate into a variety of cell types upon stimulation. They should at least express clusters of differentiation (CD) CD105, CD90, and CD73 and lack the expression of CD11b, CD79a, CD19, and human leukocyte antigen-DR isotype (HLA-DR) [9]. Interestingly, MSCs uniquely display low immunogenicity, lack the expression of the major histocompatibility complex- (MHC-) II, express low levels of MHC-I, and are not inductive to lymphocytes, which reduces their chances of eliciting an immune response upon transplantation [10]. MSCs have been successfully isolated from most tissues of the body, including bone marrow, dental tissues, adipose tissues, skin, liver, lung, umbilical cord, cord blood, and placenta [11–18]. Even though clinical studies have demonstrated remarkable properties for MSCs [19, 20], reproducible, cost-efficient, standardized, and mass production of these cells and minimization of their populations' heterogeneity are important issues that are yet to be addressed, to allow for a human clinical translational therapy [21]. Through the current review, we aim to highlight the major obstacles facing MSCs' human clinical translation and how they can be overcome.

## 2. Donor-Related Factors

MSCs' quality, quantity, and characteristics rely upon a variety of donor-related factors [22], including body mass index [23, 24], age [25, 26], gender [27], and systemic and autoimmune diseases [28–30]. The variability of MSC markers' expression in correlation with their tissue source is presented in Table 1.

### 2.1. Donor's Obesity and MSCs

Obesity could impact MSCs' characteristics and regenerative potential. Comparing adipose stem/progenitor cells (ASCs) isolated from obese and nonobese patients, a significant decrease in cellular proliferation [23, 31] and colony formation [23] of ASCs obtained from obese patients was evident. Moreover, ASCs from obese patients showed altered expression of cell surface markers, with significantly decreased expression of CD54, CD66, CD90 [23], and CD29 [31] and an increased expression of CD106 and HLA II [31], in addition to significantly lower osteogenic [23, 32] and adipogenic differentiation potentials [23], as compared to ASCs obtained from nonobese patients. This was attributed to the different microenvironment associated with obesity, including adipose tissues' hypoxia, which results in increased expression of proinflammatory cytokines. Obesity-associated adipose tissue inflammation could influence ASC multilineage differentiation [23, 33]. Moreover, obesity can alter ASC stemness and expression of stem/progenitor cell-related genes (Oct4, Sal4, Sox15, KLF4, and BMI1), aside from influencing their senescence and secretome profiles [18, 24]. Additionally, obesity could diminish ASCs' immunomodulatory properties [28]. ASCs derived from obese patients were further associated with upregulation in the expression of the inflammatory cytokines interleukin- (IL-) 1*β*, IL-6, tumor necrosis factor-alpha (TNF-*α*), and monocyte chemoattractant protein-1 (MCP-1) as compared to ASCs acquired from nonobese patients [28]. These alterations were hypothesized to be mediated through activation of protein kinase C delta expression [24].

The therapeutic potential of ASCs acquired from obese and nonobese patients was explored in mice with an experimental autoimmune encephalomyelitis multiple sclerosis model. ASCs from obese patients showed an increased expression of proinflammatory cytokines as well as stimulated the proliferation and differentiation of T-cells, resulting in a failed improvement in the multiple sclerosis-associated central nervous system inflammation disease model, indicating that obesity can negatively impact the anti-inflammatory and immune-modulatory ability of ASCs [34]. ASCs from obese patients further demonstrated significantly reduced bone formation in vivo upon implantation in critical-size calvarial defects in mice, as compared to ASCs from nonobese individuals [32].

### 2.2. Donor's Age and MSCs

MSCs' number and regenerative potential are further proposed to be largely influenced by the donor's age. Rats demonstrated an age-related decrease in bone marrow mesenchymal stem/progenitor cell (BMSC) yield [26, 35] and proliferation rate as well as a significant reduction in their osteogenic capacity in vitro [26] and in vivo following subcutaneous implantation [36]. Likewise, human BMSCs and ASCs displayed an age-related increase in cellular senescence (apoptosis) and expression of p53 gene [25] in addition to a decrease in the cellular proliferation rate [25, 37] and osteogenic [25, 37–40] and chondrogenic differentiation in vitro [37, 41], with an increase in adipogenic potential, reflected clinically by an increased adipose deposition in the bone marrow [40]. Comparing human MSCs acquired from young and old donors, an age-related decrease in cellular proliferation and increased apoptosis, attributed to p53/p21 and p53/BAX pathway activation, respectively, was observed. In addition, an increase in cells positive for senescence-associated *β*-galactosidase and a decrease in osteogenic differentiation, alkaline phosphatase (ALP), Runt-related transcription factor-2 (RUNX-2), Osterix, bone sialoprotein, and osteocalcin expressions was observed. This was attributed to an upregulation of p53 gene expression, which negatively correlates with osteoblastogenesis [25].

Interestingly, nonadherent, less differentiated rodents' BMSCs in suspension cultures appeared to be more resistant to the effect of aging in vitro [42]. Nonadherent cells showed elevated expression of pluripotency markers Nanog, Oct4, and Sox2. Further, the generation of colonies by nonadherent MSCs collected from old rats was not reduced as compared to young rats [42].

In addition to epigenetic changes leading to cellular senescence, aging of MSCs is believed to be further caused by DNA damage, telomere shortening, and accumulation of oxidative stress. All these events could in isolation or combined lead to changes in MSC cellular functions including proliferation and differentiation [43–46]. Reactive oxygen species (ROS) accumulates intracellularly in MSCs with age. Increasing levels of ROS subsequently cause oxidation of cellular components, senescence, and DNA damage, negatively influencing the differentiation ability of MSCs [47, 48]. Aging is further associated with dysregulation in micro-RNAs (miRNAs), the noncoding RNA regulating gene expression [43]. In this context, aging processes were observed to be accompanied by a decline in miR-27a associated with osteogenic differentiation [49] as well as an upregulation in miR-335 [50], miR-199b-5p [51], miR-31a-5p [52], and miR-29c-3p [53] associated with increased senescence, decreased proliferation, and osteogenic differentiation [50–53].

Senescent MSCs display changes in expression of genes associated with proliferation, signaling, function, and maintenance of MSCs, with an age-related loss in MSC response to biological signals. In addition to age-related change in DNA methylation, a reduction in expression of the transcription factors ALX1, PITX2, HOXB6, HOXB7, and IRF6 and increased expression of TBX18 and FOXP2 involved in cellular senescence, disruption in mitochondrial function, and reduction in differentiation ability of MSCs have been reported [51]. As continuous shortening of the telomeres results in reduced proliferation and differentiation, BMSCs transduced with telomerase gene maintained proliferation and differentiation potentials in vitro [54].

The effect of aging on MSCs can also be ascribed to age-associated inflammation, as levels of inflammatory cytokines especially TNF-*α* tend to increase with age [55]. TNF-*α* at high concentrations exhibited a capacity to induce MSC apoptosis in a dose-dependent manner. Additionally, its amalgamation with IFN-*γ* considerably hastens this procedure, by switching the signaling of an IFN-*γ*-activated nonapoptotic form of TNF receptor superfamily member 6 (Fas) to a caspase-3- and caspase-8-associated proapoptotic cascade, accompanied by a reduction in intracellular NF-*κ*B levels, apoptotic pathway activation, and culmination of cell death [56]. Excessive inflammation therefore appears to drive cellular senescence.

Conversely, other studies concurred that the aging process had an insignificant effect on ASC senescence and regenerative capacity [57, 58]. Intradonor comparison of ASCs collected from different donors and cryopreserved for 7 to 12 years with ASCs isolated from the same donor at a later time-point revealed a non-age-related decrease in the number of progenitor cells or proliferation rate. Additionally, cells from different timelines were capable of adipogenic, osteogenic, and chondrogenic differentiation, further denoting that the regenerative capacity of ASCs could be preserved with age [57]. Interestingly, human dental pulp MSCs collected from different age groups further displayed remarkable proliferative and differentiation abilities into bone, endothelial, glial, and neuronal cells during early passages in vitro and a potent regenerative capacity upon loading on scaffolds and implantation in rats' calvarial defects in vivo [59]. However, periodontal ligament-derived MSCs showed an age-related decrease in cell proliferation and adipogenic and osteogenic differentiation [60].

Thus, ASCs [57, 58] and dental pulp MSCs [59] could offer a convenient alternative to BMSCs for regenerative purposes in aging patients. Still, MSCs' banking from a younger age population and allogenic MSC transplantation could represent beneficial alternatives to overcome age-associated depletion in the number and regenerative capacity of MSCs [58, 61].

### 2.3. Donor's Gender and MSCs

The effect of gender on MSC regenerative abilities is still disputable. Female rats demonstrated a lower number of bone marrow progenitor cells and significantly decreased osteogenic and adipogenic potentials as compared to male rats [35]. On the contrary, BMSCs isolated from female rhesus monkeys demonstrated a higher neurogenic potential as compared to those isolated from male rhesus monkeys [27].

### 2.4. Donor's Systemic Diseases and MSCs

MSCs from patients with systemic diseases, including type II diabetes mellitus [28, 62], rheumatoid arthritis [29], and osteoarthritis [30], and from cows suffering from endometritis [63] have demonstrated altered cellular functions.

ASCs acquired from obese donors with type II diabetes mellitus showed a significant upregulation of their expression of the immune modulators IL-1*β*, IL-6, TNF-*α*, and MCP-1 as well as inflammatory regulators, including NLRP1, NLRP3, and caspase-1. They further demonstrated less ability to suppress T- and B-cell proliferation and were associated with diminished activation of the immunomodulatory M2 macrophage phenotype, indicating that obesity and type II diabetes are associated with a reduction in the immunosuppressive effect of ASCs [28]. Concomitantly, culturing ASCs isolated from both diabetic and nondiabetic patients at high glucose concentrations significantly decreased cellular proliferation, colony-forming abilities, and osteogenic and chondrogenic differentiation as well as additionally increased senescence, apoptosis, and adipogenic differentiation, with a more pronounced effect observed on diabetic ASCs [64]. Type II diabetes-associated alteration in MSCs was attributed to diabetic hyperglycemia, chronic systemic inflammation, increase in proinflammatory cytokines [65, 66], and accumulation of advanced glycation end products (AGEs) [66]. Accumulation of AGEs results in ROS production and increased oxidative stresses [65, 67].

Bone marrow MSCs isolated from patients with rheumatoid arthritis displayed a decreased proliferative and migration activity and a reduced ability to inhibit T-helper 17 cell polarization, responsible for maintaining chronic inflammation [29]. Similarly, those isolated from patients with osteoarthritis showed reduced proliferative, chondrogenic, and adipogenic potentials [30].

A significant improvement in cardiac functions with a significant decrease in myocardial apoptosis was detected in a coronary artery disease rat model following transplantation of MSCs isolated from patients suffering from coronary artery disease as compared to those isolated from patients suffering from coronary artery disease and diabetes [62]. Endometrial MSCs isolated from cows with endometritis showed a decrease in colony formation and adipogenic differentiation. Additionally, healthy cows' endometrial MSCs exposed to inflammatory mediator prostaglandin E2 in vitro displayed alteration in expression of 1127 genes related to cellular biological processes [63].

### 2.5. Inflammation and MSCs

MSCs have well-documented immunomodulatory properties. Yet, MSCs derived from chronic inflammatory environment could display different altered immunological characteristics [68]. TNF-*α* impact on MSCs depends upon dosage and exposure duration. Short-term TNF-*α* treatment has displayed a dose-dependent effect on murine MSCs in vitro. Lower doses increased osteogenic differentiation while higher doses negatively impacted MSCs and reduced osteogenic differentiation via the NF-*κ*B signaling pathway. On contrary, long-term treatment inhibited osteogenesis at both dosage regimens [69].

MSCs isolated from human calcified aortic aneurysm with chronic inflammation displayed strong osteogenic differentiation and mineralization in addition to pathologic vasculogenesis. Short-term culturing of MSCs isolated from a healthy aorta for 24 hours with TNF-*α* or IL-1*β* enhanced their osteogenic differentiation in vitro [70]. Similarly, MSCs injected into a mouse model of collagen-induced arthritis exhibiting chronic inflammatory environment were associated with dysregulation in their immunomodulatory function. Additionally, MSC pretreatment with TNF-*α* inhibited their ability to suppress T-cell proliferation in vitro, demonstrating the ability of TNF-*α* to inhibit MSC immunomodulation [71].

Periodontal ligament stem/progenitor cells derived from inflamed tissues displayed altered characteristics, with higher proliferation and migration tendency as compared to MSCs derived from healthy periodontal ligaments. They further displayed reduced immunomodulatory properties in addition to downregulation in osteogenesis-related genes (osteocalcin, RUNX-2, and ALP), while adipogenic differentiation was maintained [72, 73]. Coculturing of periodontal ligament stem/progenitor cells derived from inflamed tissues with peripheral blood mononuclear cells showed reduced ability to inhibit T-cell proliferation, T-helper 17 differentiation, and IL-17 secretion [74]. Treatment of human periodontal ligament stem/progenitor cells during osteogenic differentiation with high doses of TNF-*α* was found to be associated with downregulation in ALP, bone sialoprotein, osteocalcin, and RUNX-2 expression. The indicated inhibition of osteogenic potential denotes the negative effect of inflammatory cytokines in high concentration on osteogenic differentiation. On the other hand, BMSCs were more resistant to the inhibitory effect of TNF-*α* [75].

MSCs isolated from healthy buccal mucosa showed a higher proliferation rate and higher ability to suppress T-cell proliferation as compared to MSCs isolated from oral lichen planus lesions. MSCs harvested from lichen planus lesions further showed higher adipogenic tendency [76].

Stem/progenitor cells from healthy pulps showed a higher initial proliferation rate, as well as stronger adipogenic, chondrogenic, and osteogenic potentials, than stem/progenitor cells from inflamed dental pulp tissues. They also displayed higher expression of cell surface markers CD73, CD90, and CD166 in addition to HLA-G, involved in immunomodulation as well as stronger suppression of T-cell proliferation as compared to dental MSCs derived from inflamed pulp [77]. Further, T-lymphocytes cultured with MSCs derived from inflamed dental pulps secreted a higher amount of IL-2, TNF-*α*, and TNF-*β* [78].

Similarly, umbilical cord-MSCs treated with either interferon gamma (IFN-*γ*), TNF-*α*, IL-1*β*, IL-2, or IL-6 for 3 or 7 days presented altered phenotype and function. INF-*γ*, TNF-*α*, and IL-1*β* upregulated the expression of CD54, while TNF-*α* upregulated CD106 expression. TNF-*α* and IL-1*β* reduced the proliferation rate, while IL-6 stimulated cell migration. All inflammatory cytokines were reported to inhibit the adipogenic capacity, while chondrogenic and osteogenic differentiation capacity was enhanced by TNF-*α* and IL-1*β* coculture. Additionally, indoleamine 2,3 dioxygenase (IDO) was inhibited by TNF-*α* [79].

### 2.6. MSC Preactivation with Inflammatory Mediators

MSC preactivation (licensing or preconditioning) involves pretreatment of MSCs with inflammatory mediators including IFN-*γ*, IL-1*β*, and TNF-*α* to enhance their immunosuppressive properties and therefore increase immune-tolerance, following allogenic stem/progenitor cell transplantation [80–82].

BMSCs preconditioned with IFN-*γ* for 48 hours showed upregulated HLA-DR and IDO expression. Activation of MSCs was associated with upregulation of HLA class II and programmed death-ligand 1, which induces inhibition of T-helper cells. Activated MSCs also inhibited HLA-mismatched T-helper cell proliferation and demonstrated the ability to take up and process antigens [83]. Equine BMSCs exposed to inflammatory stimulation via preconditioning with TNF-*α*, IFN-*γ*, or inflamed synovial fluid revealed downregulated expression of migration-related genes with upregulation in adhesion-related molecules and MHC-I gene expression. TNF-*α* and IFN-*γ* were associated with dose-dependently increased expression of immunoregulatory molecules responsible for T-cell suppression, including cyclooxygenase 2, inducible nitric oxide synthase, IDO, and IL-6, in addition to upregulation of MHC-II expression [84]. Similarly, activation of ASCs with IFN-*γ* enhanced their ability to inhibit T-cell proliferation.

However, pretreatment with TNF-*α*, IL-1*β*, IL-17, tissue growth factor-*β*, or stromal cell-derived factor-1*α* did not show similar effect [85]. Treatment of MSCs from healthy buccal mucosa with IFN-*γ* was associated with the initial increase in proliferation followed by reduction in the rate of proliferation, following 12 days of IFN-*γ* treatment. Furthermore, IFN-*γ* treatment promoted MSC-mediated T-cell proliferation inhibition via IDO activity [76]. Likewise, IDO expression was upregulated upon stimulation of human periodontal ligament stem/progenitor cells by IFN-*γ* in vitro [86]. MSCs preactivated with IFN-*γ* prior to cryopreservation effectively blocked T-cell proliferation and secretion of T-helper cells promoting cytokines [87].

Preconditioning of human MSCs with IL-17 [88], IL-1*α*, or IL-1*β* [89] was also associated with a positive outcome. IL-17 effectively enhanced MSC immunomodulatory functions without increasing MHC-I or MHC-II [88]. Preconditioning of human BMSCs with IL-1*α* or IL-1*β* for 24 hours demonstrated an increase in the secretion of granulocyte colony-stimulating factor, which was not observed upon preconditioning with TNF-*α* or IFN-*γ* [89].

Thus, it can be concluded that the surrounding environment can modulate characteristics and immune-related functions of MSCs as it can either promote anti-inflammatory or proinflammatory reaction of MSCs, implicating them in the pathogenesis of multiple disorders and reducing their regenerative applications. The severity of inflammation, nature, dose, and duration of the proinflammatory cytokines govern and direct MSC reaction. Further, the differentiation capacity of MSCs under inflammatory challenge is highly influenced by the original tissue source and microenvironment of donor tissue [90–96].

## 3. Cell Source

Heterogeneity of cell sources is a further challenge for clinical applications of MSCs. Cell source heterogeneity is related to the donor (whether autograft or allograft) and the organ/tissue selected for MSC isolation [21].

### 3.1. Autogenic versus Allogenic Cell Sources

MSCs can be either acquired from the same recipient (autogenic graft) or another donor within the same species (allogenic graft) [97]. Autogenous grafting is a safe disease-free approach in MSCs' therapy [98]. However, many variables could affect autogenous cell grafting, including donors' age [25, 26], sex [27], body mass index [23, 24], and systemic autoimmune and inflammatory diseases [28–30] (discussed above), making it difficult to obtain a sufficient number of healthy MSCs without ex vivo expansion [99, 100]. The process of isolation of autogenous MSCs can further be costly and time-consuming, limiting its use in acute conditions.

Several studies endorse the utilization of allogenic MSCs instead of autogenic ones for regenerative purposes [101–103]. The low immunogenicity of MSCs encouraged the use of allogenic MSCs as they are less likely to elicit an immune reaction. MSCs are characterized by low expression of MHC-I and lack of expression of MHC-II as well as B- and T-cell stimulating antigens CD40, CD80, CD86, B7-1, and B7-2 [104–106]. Loading-induced cartilage defects in rabbits' femoral condyles with either allogenic or autogenic BMSCs were associated with an effective repair of these defects [107]. Furthermore, autogenous or allogenic ovine BMSCs loaded on scaffolds, following osteogenic differentiation and implanted in an ovine critical-size segmental defect model, showed positive results in bone regeneration, with no significant differences observed between them [108]. Similarly, positive results were attained upon allogenic MSC intra-articular injection in horses [109]. Transplantation of allogenic MSCs further showed promising results in neurogenic regeneration in a canine spinal cord injury model [110] and regeneration in a muscular dystrophy hamster model [111]. Randomized clinical trials demonstrated a potent regenerative potential of allogenic MSC administration on cardiac [101, 102, 112], hepatic [103], and cartilage [113] tissues with no adverse effects. Patients suffering from left ventricular dysfunction were randomly assigned to receive either autogenic or allogenic MSCs via transendocardial injection. Both treatments yielded equally positive outcomes with no reported undesirable side effects [101, 102]. Additionally, upon administrating bone marrow, umbilical cord, or cord blood allogenic MSCs to patients with chronic hepatic failure via intravenous infusion, clinical improvements were observed in all groups with no adverse effects [103]. Promising results were also observed in cartilage regeneration in patients with osteoarthritis [113].

In this context, commercialized allograft can provide a reproducible, readily available product with reduced cost and production time, compatible with quality standards, and good manufacturing practice (GMP), making it an efficient alternative to autogenous stem/progenitor cell therapy [99, 102]. Remestemcel-L (Prochymal) was one of the first commercial cryopreserved allogenic BMSCs used successfully for the management of graft versus host disease to be approved in Canada [114, 115]. In Japan, TEMCELL, allogenic BMSCs, was also approved for management of graft versus host disease [116]. Darvadstrocel (Alofisel), a cryopreserved allogenic ASC and the first allogenic stem cell therapy to be approved in Europe, was further used for the treatment of perianal fistulas caused by Crohn's disease [117].

However, results reported in literature regarding the impact of cryopreservation on BMSC banking are controversial. A systematic review that analyzed forty-one in vitro studies concluded that cryopreservation does not affect BMSCs' morphology and surface markers, differentiation, or proliferation potential. However, varied results exist regarding its effect on colony-forming ability, viability, attachment, migration, genomic stability, and paracrine functions. This was primarily attributed to the vast variations in the cryopreservation process and lack of standardized assays [118].

Further, it was suggested that MSCs could be immune evasive in vivo rather than being truly immune privileged as previously thought and can trigger an adverse immune response [119, 120]. Some studies demonstrated the presence of antibodies against allogenic MSCs with subsequent rejection of administered allogenic MSCs in animal models [80, 119, 121–123]. Inflammatory prestimulation of MSCs in particular could induce a negative effect, as preconditioning of MSCs was commonly associated with increased MHC expression [83, 84], stimulating an elevated antibody production, leading to subsequent adverse reactions and heightening of the immune clearance, especially following repeated allogenic stem cell transplantation. In the same context, equine BMSCs primed with proinflammatory cytokine displayed higher expression of MHC-I and MHC-II. Following intra-articular injection in the osteoarthritis equine model, allogenic primed MSCs mediated antibody production and primary humoral responses in horses with equine leukocyte antigen expression, partially compatible and incompatible with donor MSCs. Repeated MSC injection was associated with secondary humoral immune response. Although demonstrating less antibody production, these antibodies easily targeted primed MSCs because of their higher MHC expression and showed high cytotoxicity toward allogenic MSCs as compared to unprimed MSCs [124]. Thus, transplanted allogenic MSCs should be subjected to extensive characterization, and their immunogenicity should be thoroughly assessed prior to implantation.

MSC secretome was further suggested as a novel cell-free therapeutic product that recapitulates various cytokines, growth factors, extracellular matrix (ECM) proteins, and vesicles secreted by MSCs [18, 125–129]. MSC secretome might represent a clinical alternative to treat patients instantly, while overcoming the limitations and risks associated with cell-based therapy [130, 131]. Although MSC conditioned medium (CM) and extracellular vesicles have demonstrated regenerative potential in treating diseases and injuries of the nervous system, heart, lung, liver, periodontium, and soft and hard tissues [18, 132–140], several issues must be addressed before its successful clinical application, including the elimination of any xenogenic constitutions and the determination of the exact dosage, frequency of administration, protein composition, and mechanism of action [18, 131, 141].

### 3.2. Donor Tissue Source

As previously mentioned, MSCs have been isolated from multiple sources. Tissue of origin can highly impact MSCs' characteristics and differentiation ability [11]. BMSCs have superior osteogenic and chondrogenic potentials [142]. Yet, bone marrow harvesting is a rather invasive procedure [143], the percentage of mesenchymal progenitors in bone marrow is relatively low [144], and BMSCs have lower proliferation rate as compared to MSCs from other sources [145].

ASCs were originally described as a more convenient alternative to BMSCs [146] with less invasive isolation procedure [147], higher yield of progenitor cells [148, 149], and greater proliferation rate [145, 150]. The density and properties of ASCs depend on the location of the adipose tissues from which they were isolated [23, 151, 152]. ASCs isolated from visceral adipose tissue showed reduced proliferation and adipogenic and osteogenic differentiation as compared to ASCs isolated from subcutaneous tissues of the same donor [23]. Further, rats' cervical brown fat showed significantly higher MSCs' yield as compared to other locations [152]. Unfortunately, ASCs have a strong adipogenic differentiation tendency [153, 154], in addition to decreased proangiogenic factors and cytokine secretion as compared to BMSCs [155, 156].

Dental tissues further represent a potent source of MSCs, isolated via minimally invasive procedures [157, 158]. Dental MSCs include dental pulp stem/progenitor cells isolated from dental pulp tissues of permanent teeth, stem/progenitor cells extracted from pulp tissues of human exfoliated deciduous teeth (SHED), periodontal ligament stem/progenitor cells isolated from periodontal tissues, dental follicle stem/progenitor cells isolated from dental follicle surrounding the third molar, alveolar bone-derived stem/progenitor cells, stem/progenitor cells isolated from apical papilla at the apices of immature permanent teeth, tooth germ progenitor cells isolated from late bell stage third molar's tooth germs, and gingival stem/progenitor cells isolated from gingival tissues [18].

Dental stem/progenitor cells especially gingival and alveolar bone proper MSCs [157, 159, 160] can be isolated during routine dental treatments [161] and possess higher proliferation rates, as compared to either BMSCs or ASCs [162, 163]. Additionally, they have high osteogenic, chondrogenic, adipogenic, neurogenic, and angiogenic potentials [161, 164]. Even though dental stem/progenitor cells provide an appealing source for tissue regeneration, some types as gingival stem/progenitor cells may be inaccessible while others as SHED, dental follicle stem/progenitor cells, stem/progenitor cells from apical papilla, and dental pulp stem/progenitor cells may be difficult to isolate in sufficient amounts [165].

Perinatal MSCs isolated from the placenta, umbilical cord, and umbilical cord blood were further suggested to offer a noninvasive alternative source to adult MSCs. They are easily acquired, possess higher proliferative rates, and exhibit longer culture times, higher expansion, delayed senescence, and high differentiation potentials. Additionally, the placenta and umbilical cord provide a large number of progenitors as compared to MSCs from other sources [61, 166–170]. Yet, the isolation and culture of MSCs from the umbilical cord are difficult [171], while private banking of the umbilical cord and umbilical cord blood is expensive [171, 172] and lacks strict regulations [171, 173]. Moreover, the effect of lifelong storage of umbilical tissue or umbilical cord blood is still unstudied [171, 174, 175]. The major problem associated with the application of umbilical cord blood remains to be the limited amount of cells extracted from each donor as cord blood volume is limited [176], where a single umbilical cord blood unit contains 50 to 200 ml of blood [177]. Umbilical cord blood yields a much lower amount of MSCs as compared to BMSCs [178]. It also has slow engraftment as compared to BMSCs [173]. Placental MSCs further carry a safety hazard regarding possibility of contamination during placenta collection and possible tumorigenic transformation [179].

## 4. MSCs' Isolation Procedures

MSC isolation from different tissues is one of the most critical steps prior to their ex vivo preparation, greatly impacting their quality and quantity [61]. For clinical applications, great attention should be given to the selection of the proper method of isolation [180]. Challenges facing MSC isolation are related to different factors, including the presence of various isolation protocols, the diverse MSC sources, and the fact that MSCs are usually present in very minute concentrations in their respective tissue sources [61, 150]. Although MSCs possess unique properties and have a great potential for clinical application, up to date, no exclusive set of markers exists for their identification and isolation [181]. Hence, there is currently a mandatory demand to increase the minimal criteria proposed by the International Society for Cellular Therapy in 2006 for MSC identification [9], to encompass the inclusion of paracrine factors or immunomodulatory properties of MSCs [182] as important predictors for their success during clinical application [183]. Furthermore, discovering unique markers for MSC isolation with high purity is a prerequisite for developing reliable and reproducible protocols for clinical application [184, 185].

Currently, the different categories of available techniques for MSC isolation from heterogeneous cell populations depend on their unique cellular properties, including surface charge and adhesion, cell size, density, morphology, and physiology in addition to surface markers [186]. There are various categories of cell isolation techniques, namely, enzymatic, mechanical, explant culture, and density-gradient centrifugation methods [61] (Table 2).

The enzymatic method, one of the commonly used approaches, digests the tissue especially their ECM using one, two, or in some protocols three proteolytic enzymes. The differences between the several protocols described for this method include variations in the concentrations of the used enzymes, number of washing steps, centrifugation parameters, and filtration procedures [187, 188]. The efficiency and viability of the cells acquired through the enzymatic method depend on the concentration and type of the used enzyme [189–191]. Digestion periods over five minutes can affect MSCs' surface antigens [192] and cytoskeletal component, disrupt intramembranous particles, and change cell surface topography [193], which negatively affects the quality of the isolation process. Combining the enzymatic method with mechanical dissociation revealed a 70% increase in the cell yield as compared to the enzymatic method alone [194]. To overcome the problems associated with the enzymatic method, mechanical methods were introduced, using different forces such as shear, radiation, centrifugation, and pressure. Although great efforts were put into standardizing the mechanical methods, these nonenzymatic methods are still variable according to the used protocol [188].

The explant culture represents the earliest technique for cell isolation and in vitro cultivation. The tissue is cut into small fragments about few millimeters in size to facilitate nutrient delivery to the cells, avoiding excessive cutting, which may cause mechanical destruction to the cells. Following dry adhesion to the plastic culture dishes, cells start to migrate out of tissue fragments and adhere to the culture substrate surface. Subsequently, tissue fragments can be removed [11, 77, 90, 91, 158, 195–197]. The explant method demonstrates a more homogenous cell population, higher cell viability, and increased cell proliferation rates and avoids enzymatic damage as compared to the enzymatic method [198–200], which could be attributed to the gradual transition of cells from in vivo to in vitro condition [180, 201]. Comparison between ASCs isolated by either enzymatic or explant methods reveled a simultaneous expression of surface markers CD73, CD90, and CD105, as well as the absence of CD14, CD31, CD34, and CD45, making ASCs isolated by both techniques phenotypically and functionally equivalent [202]. However, the explant method depends primarily on the manual skills of the operator, which makes this method difficult to be standardized, in addition to the risk of contamination, which could affect the MSC clinical application [61].

The density-gradient centrifugation method depends on the physical and chemical parameters of the isolated cells like size, density, and hydrophobic properties. In this method, the cells move and accumulate in a position that matches the density of the medium or at the interphase in case of using two solutions with different densities [181]. Lack of high resolution in separating MSCs from other cells remains the most important limitation of this method as there is no absolute difference in size between cells [203]. Consequently, this method is mainly used as a primary step for MSC enrichment and is followed by the explant method or other higher resolution techniques such as fluorescence-activated cell sorting (FACS) and magnetic-activated cell sorting (MACS) [204, 205].

Cell isolation techniques based on antibody binding are widely advocated for the purification of MSCs with high resolution. Among the most commonly used antibody-mediated cell isolation techniques are FACS and MACS. Both FACS and MACS basically share the same idea. In the case of FACS, antibodies are linked to a fluorescent dye, while in MACS they are linked to magnetic beads and only the antibody bounded cells are separated [206, 207]. The greatest challenge for these methods of isolation remains however to be the lack of an exclusive marker of identifying MSCs [181]. Further limitations include the probability for cell contamination during sorting procedures, physical stresses exerted on the cells [208], and the dependence on adherent cell purification, where the use of enzymes for cell detachment can cause proteolytic damage to cell surface proteins [61]. Some of those concerns were postulated to be overcome with the development of the CliniMACS Cell Isolation System, a device that is currently clinically approved and takes advantage of conjugating colloidal suspension of superparamagnetic microbeads to a monoclonal anti-human antibody that is capable of binding to its antigen in bone marrow, umbilical cord blood products, and peripheral blood in a sterile GMP system [209].

Recently, the emergence of different isolation methods changed the typical pattern of adherent MSCs and provided another source of MSCs known as “nonadherent cell population” (NACP) [210, 211]. These NACP were obtained during medium exchange of marrow MSC culture, where the floating cells were centrifuged and replated in separate flasks. Surprisingly, these cells revealed the same proliferation and differentiation potentials as the originally attached MSCs in vitro [210]. Likewise, NACP isolated from fat resources demonstrated similar proliferation and differentiation potentials as MSCs [212]. These findings demonstrated that NACP could be a simple method to enrich MSCs' number for clinical application.

Choosing the proper MSC isolation method depends mainly on certain features that should be compared between the different available techniques, including cell purity, cell recovery rate, cell yield, and cell viability [186, 213]. Moreover, the selected technique should be minimally invasive, rapid, and with high-resolution quality [214]. Therefore, for successful clinical translation of MSCs, a well-established method for cell isolation is a mandatory step to ensure the quality of these cells.

## 5. Cell Culture Procedures

The first challenge following MSC isolation is that their number in the primary culture without a subsequent lengthy ex vivo expansion would usually be insufficient for an immediate clinical application. Therefore, cell expansion is essential to generate a clinically appropriate number of MSCs, keeping in mind that the efficacy and safety of clinically applied MSCs are dependent on such bioprocessing procedures [215]. Thus, optimizing culture conditions to generate MSCs that retain proliferation, differentiation, and regenerative properties is one of the greatest challenges that face MSC translation to clinical application. Currently, several cell culture variables, such as the number of passages, cell seeding density, culture surface substrate, medium formulation, and the physiochemical environment in addition to different subculture protocols, are being studied [216].

### 5.1. Cell Expansion

MSC expansion could be affected by the age of MSC donors, where MSCs from young donors can undergo a higher number of population doublings in comparison to older MSCs before reaching replicative senescence [217] (discussed above). Due to this phenomenon, during the first two to three weeks of early passages, MSCs grow at a constant rate, while with the increasing number of passages, an increase in the cell doubling time until the growth stops due to senescence is observed [218]. This “replicative senescence” is caused by progressive shortening of telomere upon cell passaging in vitro due to the absence of telomerase activity [54, 219]. Despite the fact that 70–80% confluence is the recommended cellular density before passage, the decision is operator-dependent [61]. It was found that upon prolonging the MSC expansion for 43-77 days, cells demonstrated senescence features, including abnormality in morphology, arrested proliferation, decreased expression of cell surface markers, loss of differentiation capacity [220], and decrease in their capacity for migration [221]. Furthermore, prolonged cultivation of MSCs may cause chromosomal changes, which could predispose for malignant transformation [222]. It has been reported that upon comparing human umbilical cord-MSCs at passages 3, 6, and 15, the cells showed similar morphology, biomarker expression, and cytokine secretion. At passage 15, despite the fact that the cells were still potent regarding adipogenic differentiation and cytokine secretion such as IL-6 and VEGF, they revealed inferior cell proliferation ability and less osteogenic and chondrogenic differentiation potentials. Moreover, human umbilical cord-MSCs at passage 15 revealed impaired hematologic supporting effect in vitro and declined therapeutic potential on a GVHD in vivo [223].

To overcome cellular senescence, MSCs could be genetically modified by a retroviral vector containing the gene for the catalytic subunit of human telomerase reverse transcriptase (TERT). Transduced cells (MSCs-TERT) demonstrated telomerase activity, with the ability to undergo more than 260 population doublings, in contrast to nontransduced control cells, which underwent senescence-associated proliferation arrest after 26 population doublings [54]. Upon subcutaneous implantation in immunodeficient mice, MSCs-TERT formed more bone as compared to their controls. However, in a further study, MSCs-TERT showed loss of contact inhibition and anchorage independence and lead to tumor formation in all mice [224]. Therefore, although considering intermittent activation of the TERT gene may be an interesting approach, it may be linked to dangers related to tumorigenicity.

On the other hand, telomerase activation was found to influence the MSC regulatory path, where ectopic expression of the TERT gene in human postnatal BMSCs sustained their osteogenic potential and upon xenogenic transplantation formed more bone tissue with a normal structure as compared to the control human postnatal BMSCs [225]. This enhancement was attributed to the high expression of early preosteogenic stem cell marker STRO-1, which revealed that telomerase expression assists in maintaining the osteogenic potential of MSCs during their expansion.

Moreover, the differentiation potential of an immortal adipose stromal cell line (ATSC) transduced with a retroviral vector expressing TERT was assessed in vitro [226]. ATSC-TERT cells significantly accumulated calcium one week after being cultured in osteogenic induction medium, while control ATSC cells began to accumulate it after three to four weeks. Additionally, the expression of osteoblastic markers (osteoblast-specific factor 2, chondroitin sulfate proteoglycan 4, and TNF receptor superfamily) was increased in ATSC-TERT cells as compared to control ATSC. The insulin-like growth factor (IGF) signaling pathway especially, IGF-induced AKT phosphorylation, and ALP activity were postulated to be involved in the mechanisms through which the TERT gene enhances osteoblastic differentiation [227].

Another important factor to consider during MSC expansion is the prior usage of proteolytic enzymes for cell detachment during the expansion process. Proteomic results revealed differential expression of 36 proteins in trypsin-treated cells and an upregulation of the expression of proteins related to apoptosis, with downregulation of proteins related to cell growth, cell adhesion, regulation of metabolism, and mitochondria electron transport [228]. Three-dimensional (3D) culture systems may be the solution to overcome all the limitations associated with MSC expansion, as it could allow their propagation without the use of proteolytic enzymes [229]. Consequently, great attention should be given to 3D culture systems to standardize their effect on MSCs.

### 5.2. Cell Seeding Density

Cell seeding density impacts cell proliferation, differentiation, and ECM formation [230–232]. BMSCs seeded at lower density (100 cells/cm^2^) possessed a faster proliferation rate than those seeded at higher density (5000 cells/cm^2^) [233]. Moreover, high cell seeding density of (10^6^ cells/cm^2^) caused a minimal increase in the cell number in comparison to lower seeding density on 3D scaffolds [234]. The low growth rate of MSCs seeded at high densities could be attributed to contact inhibition, while a higher growth rate associated with low seeding density could be attributed to the presence of the small and agranular cells (recycling stem cells) in the log phase. Those cells are postulated to give rise to large numbers of cells during the log phase of exponential growth [235]. The log and exponential phases last for longer duration in cells seeded at low density, and therefore, more population doublings occur [236]. Unfortunately, there is a limitation of low initial seeding density as it has been reported that BMSCs platted at 10-100 cells/cm^2^ did not expand effectively and the cells were senesced after four to five passages [237]. Although low seeding densities revealed higher proliferation rates, it is unrealistic for large-scale clinical MSC production as the needed number of culture flasks exceeds the manageable limit of practical handling and cost-effectiveness [238].

The cell seeding density affects the stemness gene expression and senescence of MSCs, where lower density seeding (200 cells/cm^2^) of ASCs caused upregulation of stemness genes Oct4, Nanog, SRY-box 2, KLF4, c-Myc, and lin-28 homolog A, especially Nanog and c-Myc in comparison to high-density seeding (5000 cells/cm^2^) [239]. Moreover, it was reported that the optimal cell growth of BMSCs could be achieved at a plating density of 200 cells/cm^2^, with no differences observable in their differentiation potential at different densities (20, 200, and 2000 cells/cm^2^) up to 5 passages [236]. It was further demonstrated that high cellular seeding density (5 × 10^6^ cells/ml) of BMSCs on collagen microspheres favored chondrogenic differentiation [240]. Comparing dental pulp MSCs cultured under sparse (5 × 10^3^ cells/cm^2^) and dense (1 × 10^5^ cells/cm^2^) seeding conditions for four days revealed observable enhancement in mineralized nodule formation in densely plated dental pulp MSCs [241]. In addition, densely plated dental pulp MSCs demonstrated more pronounced mineralized tissue formation in comparison to sparsely plated dental pulp MSCs when implanted into mouse bone cavities [241].

These findings suggest that cell seeding density could favor the differentiation of MSCs toward specific cell lineages. Determining the optimum cell seeding density designed for maximum cell expansion is therefore of great significance for clinical application, as it could shorten the cell culture time and consequently decrease the risk of culture contamination and alteration in the MSC characteristics [242].

### 5.3. Culture Media

Choosing a well-formulated culture medium for expansion and therapeutic application of MSCs is very crucial [243]. A typical culture medium is composed of amino acids, vitamins, glucose, inorganic salts, and serum [244]. Culture media can affect MSCs' secretion profile. Studies deduced that cytokine and growth factor secretion is donor-specific [125] and that cellular passaging does not significantly influence MSCs' secretome properties [18, 245], while other investigations demonstrated that the cell culture medium might affect the MSC secretory potential to varying degrees [246, 247].

Among the commonly used basal medium formulations for culturing of human MSCs are Dulbecco's modified Eagle's medium (DMEM) and alpha minimal essential medium (*α*-MEM). Although DMEM was widely used for MSC expansion [248–252], later it was demonstrated that *α*-MEM could show better performance in isolation, expansion [253], and osteogenic induction of MSCs [233] as primary dental pulp MSCs [241]. MSC differentiation into various cell types could be achieved by adding certain substrates to the culture media. Osteogenic differentiation could be mediated by *β*-glycerophosphate and ascorbate phosphate; adipogenic differentiation could be induced by isobutyl-methylxanthine and indomethacin, while chondrogenic medium usually contains transforming growth factor-*β* (TGF*β*1) and ascorbic acid [254, 255]. Neural differentiation was achieved in media supplemented with both epidermal growth factor (EGF) and fibroblast growth factor- (FGF-) 2 [256, 257], while hepatic differentiation occurred in media supplemented with hepatocyte growth factor (HGF), bFGF, and oncostatin [255].

Basal media do not contain proteins or growth-promoting agents and therefore require supplementation with fetal bovine serum (FBS), typically 10% to 20% [61]. FBS is the most excessively used serum in cellular culture procedures, as it provides important elements such as nutrients, hormones, growth factors, and carrier proteins. These carrier proteins encompass hormones, vitamins, attachment and spreading factors, lipids, metals, protease inhibitors, and buffering agents, whose cumulative function is to back up cellular growth [258]. A number of successful clinical trials were conducted utilizing MSCs expanded in FBS-containing media [259, 260].

Yet, the usage of animal-derived serum is not the best choice for clinical applications, due to the risk of the possible transmission of nonhuman infectious pathogens such as viruses, prions, mycoplasma, and endotoxins [261–270]. Furthermore, the high content of xenogenic antigens in FBS could elicit an immune response in recipients following MSC transplantation [268, 269, 271, 272]. Moreover, lack of uniformity in the composition of serum between different companies and the high degree of lot-to-lot variation in terms of growth factor concentrations [263, 273] contribute to the heterogeneity of the results following MSC transplantation [273, 274]. Thus, before utilization, regular testing could be needed in order to ensure the quality of each batch, an additional obstacle that hinders the fabrication of an MSC-based standardized product [216].

The presence of serum in media may interfere with the purification and expansion of cell culture products since it could contain growth-inhibiting factors as fetuin (*γ* globulin) and growth-promoting factors that occasionally could inhibit cell growth depending on their concentration and the stimulus-response decisions made by the stem/progenitor cells [275]. These growth factors include but not limited to platelet-derived growth factor (PDGF), IGF, and EGF [276] in addition to TGF*β*, which regulates the actions of many other signaling molecules. TGF*β* was documented to inhibit the growth of mouse keratinocytes [277], while EGF was reported to inhibit human epidermoid carcinoma cells [278]. The diversity of these factors might lead to clinical complication and data misinterpretation [244] (effects of different growth factors are discussed later in MSCs and Growth Factors).

In order to consider MSCs as an advanced therapy medicinal product, serum-free media have been proposed to attain large-scale quality and relatively low-cost production of clinical-grade MSCs [279, 280]. These medium formulations incorporated defined quantities of binding proteins (i.e., albumin and transferrin), additional nutrients (i.e., lipids, vitamins, and amino acids), physiochemical reagent (i.e., buffer), hormones (i.e., insulin), growth factors (i.e., EGF, PDGF, and FGF), and attachment factors [281, 282], which are all usually provided by the serum. The optimization of defined serum-free medium for a specific cell type is very difficult and influenced by multiple variables regarding cell characteristics, FDA-approved serum-free/xeno-free culture media as an example for such substitutes [283–286].

An ideal FBS alternative for clinical GMP production should possess a well-defined composition, a reduced degree of contaminants, no risk of xenogenic compound transmission, low production costs, easy availability, and no ethical issues [250]. Using autologous or allogenic serum, plasma, or platelet lysates was further proposed for cultivating and expanding human MSCs [280, 287], although it may be difficult to attain sufficient amounts from these substrates. Moreover, their beneficial effect may decrease with age, becoming nonapplicable in elderly patients [61]. Furthermore, autologous or allogenic serum may not contain sufficient growth factors to support the growth of MSCs [258].

Human platelet lysate (hPL), prepared by lysis of the platelet membrane, was found to meet most of these requirements and was suggested as a natural reservoir of growth factors and cytokines such as basic FGF, EGF, HGF, IGF-1, PDGF, TGF*β*1, and vascular endothelial growth factor (VEGF) [259, 288], which conjointly have a positive influence on MSC proliferation and differentiation [289]. Despite this growth factor-enriched milieu, it has been reported that MSCs cultured with hPL did not express differentiation markers and differentiation only occurred upon induction [290], in contrast to media supplemented with serum, where unplanned differentiation might occur [279]. hPL can be easily obtained from autologous peripheral blood in large quantities and with minimal donor site morbidity [291]. hPL has been successfully utilized for MSC expansion in numerous in vitro studies [170, 292–300] overcoming most of the challenges associated with FBS. The composition variability, which is donor-related, may be reduced by pooling harvests of fresh blood from different donors [297, 301]. Despite its rare occurrence, the possible transmission of human diseases caused by viruses, as HIV-1 and HIV-2 or hepatitis C, can be hindered through sterilization processes employing short-wave ultraviolet light [302]. Several studies have been published evaluating the use of hPL or other xeno-free supplements for MSC ex vivo expansion, following GMP protocols [303–306]. The substitution of FBS by hPL has been reported to increase cell proliferation without affecting MSC immunophenotype, immunomodulatory potential, differentiation potential, and relative telomere length [306]. Similar results were attained when comparing two serum-free (xeno-free) media (*α*-MEM and DMEM) supplemented with 10% of hPL with DMEM supplemented with 20% FBS and 10 ng/ml bFGF. The highest proliferation rate was detected in *α*-MEM supplemented with 10% hPL [307]. It has been reported that hPL and predefined serum-free media increased the proliferation of BMSCs and ASCs [249, 285, 296]. Human umbilical cord-MSCs expanded in serum-free media propagated more slowly and were different in growth rate, telomerase, and gene expression profile from human umbilical cord-MSCs expanded in serum-containing media, yet they remained their multipotency and their therapeutic potentials [308]. On the other hand, umbilical cord-MSC expanded in hPL revealed enhanced proangiogenic and bone formation features, upon implantation combined with collagen microbeads in an immune-competent mouse model [309].

The high proliferation rate attained by hPL can reduce the MSC manufacturing time and accelerate the production of MSCs in therapeutic application. The effect of hPL on the immunomodulatory attributes of MSCs remains controversial and needs to be further evaluated, as some researchers claimed that hPL-expanded MSCs exhibited diminished immunosuppressive properties [292, 310], while others reported that hPL maintain these immunosuppressive properties [250, 311]. These discrepancies could be attributed to the differences in the hPL production assay.

In order to identify the effect of culture “micromilieu” on the critical stemness properties that could influence MSC clinical performance, dental pulp MSCs and alveolar BMSCs were cultured in two commercially available serum/xeno-free GMP culture systems (StemPro (Life Technologies); StemMacs (Miltenyi Biotek)), in comparison to conventional FBS supplemented media. Prolonged expansion of both MSC types especially in the serum/xeno-free-expanded BMSCs resulted in downregulation of CD146, CD105, Stro-1, SSEA-1, and SSEA-4, as well as in an increase of SA-gal-positive cells, cell size, and granularity and a decrease in telomere length. Moreover, expansion under serum/xeno-free systems caused an upregulation of osteogenic markers and elimination of chondrogenic and adipogenic markers while only minor changes were detected with serum-based media. Dental pulp MSCs in serum-based and StemPro revealed a diminishing mineralization potential with passaging, while with StemMacs, the opposite occurred [312].

The development of a completely defined media that lack any biological products from animals is the ultimate goal in cell-based therapy. Although serum-free media containing growth factors are postulated to maintain the main phenotypic and functional characteristics of MSCs, they are currently still inferior to FBS-containing media. hPL, which to date meets the GMP guidelines, could provide hope in this perspective.

### 5.4. Two-Dimensional (2D) Culture Systems

Conventionally, MSCs are propagated as a monolayer in two-dimensional (2D) plastic culture plates. 2D culture techniques have been developed for establishing primary cultures, cell lines, and different analytical assays [313]. In addition, 2D cultures are used for MSC differentiation into many specialized cells [314, 315].

However, the 2D culture system possesses several limitations. The first limitation of the 2D culture system is the need for cell expansion to increase the cell numbers for clinical applications. Expansion in 2D cultures is highly inefficient and yields heterogeneous populations of MSCs [316]. Moreover, 2D culture systems cause changes in cell shape [317], flattening of cells with alteration of the internal cytoskeleton and the shape of the nucleus [318], which could subsequently affect the gene expression [319, 320] and change the cell fate as well as the differentiation potential [254, 321, 322]. Within the 2D culture system, MSCs tend to undergo nonspecific differentiation where MSCs may partially differentiate or dedifferentiate with loss of functionality [316]. Besides, 2D culture conditions fail to mimic the living physiology or the in vivo MSC niche [323]. The 3D microenvironment is responsible for determining MSC fate in vivo, where it allows interactions between MSCs, ECM, and gradients of oxygen, nutrients, and byproducts [324]. In order to overcome all of these limitations, 3D culture systems have been developed to mimic the ECM composition and stiffness in vitro to control MSCs' fate [318, 324–326].

### 5.5. Three-Dimensional (3D) Culture Systems

**Figure 1:**
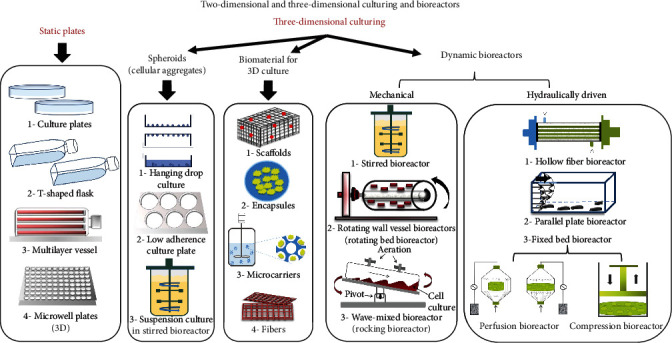
Two-dimensional and three-dimensional culturing plates and bioreactors.

In order to imitate the in vivo MSCs' niches, maintain the MSCs in their undifferentiated stem/progenitor cellular status, induce their differentiation into particular tissue for regenerative purposes, or expand them for industrial usage; various 3D culture systems have been proposed and developed. 3D culture systems vary from simple cellular aggregates (spheroids) to complex systems using dynamic bioreactors with incorporated biomaterials (Figure 1).

The spheroids allow cell-cell and cell-ECM interactions without any additional substrates [327]. These spheroids could be prepared by different techniques, including hanging drop, rotating culture, or low-adhesion culture plates in suspension culture and microwells. Human amnion mesenchymal stem cells (hAMSCs) were cultured in 2 ml of culture medium (5 × 10^5^ cells/ml) in a suspended state in a 6-well ultralow attachment plate to allow spheroid formation. The viability, multipotency, and the secretory ability for angiogenic and immunosuppressive factors were upregulated in hAMSC spheroids kept in the 3D culture system as compared to those maintained in 2D cultures. Moreover, an improved paracrine effect was recorded in vitro in the form of an increased capillary maturation as well as greater inhibition of peripheral blood mononuclear cell proliferation in the presence of 3D conditioned media as compared to both 2D conditioned media and 2D exosomes [328].

Static culture plates (culture dishes, T-shaped flasks) that are ordinarily used in 2D cultures can be modified to be dynamic to allow spheroid formation. A scaffold-free 3D culture sphere was attained upon seeding periosteum-derived progenitor cells on nonadhesive culture dishes and cultivating them at a rotation rate of 60 rpm using an orbital shaker. The resultant spheres maintained their viability and proliferation ability. Expression levels of stemness genes and proteins were upregulated in cells grown on 3D culture as compared to 2D culture systems [329]. Being heterogeneous in nature, spheroids are employed in studying cell differentiation and cancer biology [327, 330]. Upon short-term culturing, spheroids improved the medicinal properties of MSCs [331], while in long-term spheroids, culturing MSCs underwent differentiation [332].

An upregulated expression of chondrogenic genes (ACAN, COL2B, COL10, SOX9, and 18S) was recorded upon in vitro 3D culturing of equine MSCs for 4 weeks in alginate, fibrin 0.3% alginate (FA), and pellet culture systems (2.5 × 10^5^ cells and 5 × 10^5^ cells) [333]. Furthermore, the immunomodulatory characteristics of MSCs cultured in 3D culture systems constructed using collagen, chitosan, and PLGA substrates were shown to be enhanced and affected by the 3D geometry not the type of the substrate. MSCs under 3D culture demonstrated a higher growth rate and stemness and maintained their phenotype and an enhanced immunosuppression effect [334].

Yet, the wide-range growth of MSCs using these methods is challenging due to the incapability of controlling their size, leading to cell death and suppression of cell propagation as a result of a high degree of confluence and nutrient deprivation [331]. Moreover, transport and removal of nutrients and waste metabolites, respectively, from the scaffold upon 3D expansion represent a crucial obstacle. The latter process occurs in the 2D culture systems simply by diffusion [335]. While static bioreactors are limited by the demand for batch medium changes, dynamic bioreactors can be highly governable, permitting better homogenous media and cell spatial distribution, despite the increase of the scaffold. Thus, dynamic bioreactors can be utilized in tissue-engineering applications to alleviate problems related to traditional static culture conditions [336].

Incorporation of natural and synthetic biomaterials in the culture could supply diverse biological signals and allow different degrees of mechanical strength [337]. Biomaterials are utilized in the 3D culturing for fabrication of microcarriers, capsules, fibers, and scaffolds. Scaffold constructs provide the ECM 3D organization and multicellular complexity [338, 339]. Yet, natural biomaterials are more difficult to control in vitro as they often transduce uncontrollable biological signals to the cells. Moreover, the batch-to-batch variability and the potential xenogenic origin might limit their usage [340].

Dynamic bioreactor culture systems, in which the culture variables such as pH, temperature, oxygen, and carbon dioxide concentration are properly controlled and monitored, are essential for in vitro cultivation and maturation of tissue-engineering grafts [341]. These closed systems maintain a homogeneous physicochemical environment required for culturing cells and reduce the handling steps, hence reducing contamination potential in accordance with GMP and quality standards [342]. The generated hydrodynamic stress on the cells could be alleviated through utilizing biomaterials in the form of microcapsules or microcarriers [316]. Microcarriers are small beads (100–300 *μ*m diameter) that provide a surface for the cells to attach and grow while microcapsules are semipermeable membranes within which the cells are immobilized. Microcapsules allow the diffusion of nutrients, oxygen, and growth factors essential for cellular growth [316]. The selection of an appropriate biomaterial for the fabrication of either microcapsules or microcarriers as well as harvesting cells from them is among the challenges in the 3D cultures.

A rotary cell culture system (RCCS) combined with 3D culture was suggested to provide an effective means for enhanced MSCs' proliferation in vitro and to maintain a differentiation potential required for tissue engineering. The microarray analysis of BMSCs cultured in the RCCS-3D system revealed an enhanced proliferation and colony formation, as well as maintained the differentiation potential when compared with conventional static 2D and static 3D culture conditions [343].

Dynamic bioreactors (fully reviewed in articles [341, 344, 345]) could be classified into mechanically driven bioreactors that include stirred tank bioreactors, rocking bioreactors, and rotating wall vessel bioreactor, as well as hydraulically driven bioreactors, which include parallel plate bioreactors, hollow fiber bioreactors, and fixed-bed bioreactors that can be modified to perfusion and compression bioreactors widely used in bone tissue engineering (reviewed in articles [341, 346]).

Spinner flasks and stirred tank bioreactors are the most frequently used stirred systems. In these systems, impellers are used to promote mixing, resulting in a homogeneous culture system with operation versatility (batch, fed-batch, and perfusion). A large number of cells could be produced in just one vessel, thereby avoiding vessel-to-vessel variability and minimizing costs related to labor and consumables [280]. MSCs aggregated using static microwell plates prior to being inoculated in the bioreactor environment preformed controlled size aggregates possessing the ability to form large, irregular super aggregates after a few days of suspension culture. On the contrary, single MSCs inoculated directly into suspension bioreactors formed a more uniform population of smaller aggregates after a definite culture period of eight days. Both techniques showed initial deposition of ECM within the aggregates [347].

A rocking (wave) bioreactor consists of a disposable plastic bag placed on a platform whose agitated fluid motion induces the formation of waves that subsequently provide good nutrient distribution and excellent oxygen transfer with moderate shear stress. It also presents a minimum risk of contamination (closed system), scalability (up to 500 l), and flexibility [344]. No difference in differentiation and immunomodulatory capacity as well as no genetic aberrations was displayed upon culturing MSCs in flasks, Scinus bioreactor (rocking bioreactor), and spinner flasks. MSCs cultured within the Scinus bioreactor system showed equality to flask-expanded cells with respect to their immunomodulatory properties [348].

A hollow fiber bioreactor is advantageous in culturing MSCs due to its relatively homogeneous culture environment and low shear stress. The cells are inoculated within the fiber, while the culture medium flows and wastes diffuse through the pores of the fibers to the space between the cylinder and the fibers [344]. The secretory products (exosomes) of MSCs cultured using hollow fiber and their therapeutic efficacy in a murine model of cisplatin-induced acute kidney injury (AKI) in vivo and in vitro have been investigated. In vivo, both 2D- and 3D-exosomes significantly alleviated cisplatin-induced murine AKI evidenced by improved renal function, attenuated pathological changes of renal tubules, reduced inflammatory factors, and repressed T-cell and macrophage infiltration; however, the 3D-exosomes were superior to the 2D-exosomes. Furthermore, 3D-exosomes were efficiently captured by tubular epithelial cells, thereby improving their viability and inducing an upregulated anti-inflammatory effect in vitro [349].

The rotating wall vessel (RWV) and a rotating bed bioreactor (RBB) consist of a cylindrical vessel rotating horizontally around its axis. This environment eliminates most of the disruptive shear forces associated with a conventional bioreactor, randomizing the gravitational forces acting on the cell surface and allowing the combined culture of several cell/scaffold constructs [341, 350]. Collision of scaffolds with the bioreactor wall is a major disadvantage of the RWV system and may damage the scaffolds and disrupt the seeded cells. This can be alleviated by using the RBB concept, where constructs are attached directly on the axis. Another crucial disadvantage of this rotating system is that the mineralization is confined to the outer part of the scaffold upon use in bone tissue engineering and that the internal nutrient transport is deficient [346]. Thus, rotating wall vessels are limited to the small-sized constructs of flat bones or as bone patches for restorative applications of the skeletal system [351].

In bone tissue engineering using MSCs, shear stress caused by mixing or perfusion of the medium is crucial for osteogenesis, as it exposes the cells to mechanical stimulation. In vivo, mechanical stimulation increases the production of prostaglandins, ALP, and collagen type I, creating a milieu required for osteoblastic proliferation and mineralization [352]. Moreover, mechanical stimulation encourages the cells to produce ECM in a shorter time period in vitro and in a more homogeneous manner than in static culture [353].

A fixed-bed bioreactor consists of a column (bed) holding an immobilized scaffold, where the cells are incorporated. As the cells remain immobilized on the carrier surface, this system has an advantage of presenting a low shear stress environment. Although this bioreactor allows 3D cell growth and better imitation to in vivo conditions, spatial cell concentration gradients may occur [354]. Modifying the fixed-bed bioreactor has been performed to overcome the poor perfusion of media through the center of the scaffold. Bioreactors that use a pump system to perfuse media directly through a scaffold are known as perfusion bioreactors [355]. Flow perfusion bioreactors have been shown to provide more homogeneous cell distribution throughout the scaffolds and provide a uniform mixing of the media, enabling better control of the environment and better physical stimulation of the cells particularly in the bone tissue [335, 356]. The major challenges in these systems are the design of the perfusion chamber and optimization of the flow rate, which depends on the composition, porosity, and geometry of the scaffold [335]. Despite the fact that the increase in the flow rate leads to an increase in the deposition of the mineralized matrix, it seems that the optimal flow rate values have an enhanced positive effect on osteoblastic differentiation, ECM deposition, and distribution range from 0.2 to 1 ml/min [335]. Yet, perfusion bioreactor is suggested to be the ideal ex vivo culturing system for growing large bone grafts [357].

Compression bioreactors that provide mechanical loading, combined with flow perfusion, can also promote survival and functional cellular differentiation within the scaffold. Short-term mechanical stimulation enhanced the expression of several osteogenic genes, including RUNX-2, osteopontin, integrin-*β*1, TGF*β*R1, SMAD5, annexin-V, and PDGF*α* [351]. The compression bioreactors provide a promising tool for bone fracture tissue engineering [341].

Overall from the previous section, it could be deduced that although the 3D culturing maintained or even improved the therapeutic potential of the MSCs, the complexity and the diversity of these systems in terms of selecting the appropriate biomaterial to be used and bioreactor design make them additional challenges in cell-based therapy.

### 5.6. Mimicking the Biological Interactions in the Human Body

Mimicking the in vivo microenvironment to attain efficient proliferation and secretion of soluble paracrine factors and extracellular vesicles could be further achieved by hypoxia (2% O_2_) that enhances stemness in MSCs, without affecting their multipotent differentiation potential [358]. Hypoxic preconditioning (2% O_2_) of adipose-derived MSCs upregulated the proliferative ability of MSCs by enhancing the expression of normal cellular prion protein and inhibited oxidative stress-induced apoptosis via inactivation of cleaved caspase-3 in vitro. Similar results were attained upon treating a murine hindlimb ischemia model with hypoxic adipose-derived MSCs. Enhanced functional recovery of the ischemic tissue, including limb salvage, neovascularization, and the ratio of blood flow perfusion, was reported [359]. Moreover, BMSC hypoxic pretreatment enhanced significantly cell survival and promoted angiogenesis in the lower limb of ischemic diabetic rats through increasing autophagy and significantly decreasing apoptosis [360]. Additionally, hypoxic conditions increased the release of MSC exosomes that effectively enhanced the regeneration of cardiac tissues in a myocardial infarction mouse model [361].

Coculturing of MSCs with other cells could provide a promising aspect in regenerative medicine, through providing the signaling molecules, including growth factors and cytokines involved in the cross-talk between cells. In a recent study using hybrid human umbilical vein endothelial cell/rat MSC cocultures, the role of each cell type on the genes and proteins regulating angiogenesis, including VEGF, PDGF, and TGF*β*, was investigated. It has been reported that MSCs inhibited the expression of angiogenic factors in endothelial cells early in cocultures due to juxtacrine signaling-mediated suppression of cell proliferation, while later on, a shift occurred, where the restrained action of MSCs reverts to a stimulatory one by paracrine signaling. The ratio 3 : 1 endothelial cells/MSCs induced the strongest upregulation of the angiogenesis pathway [362]. Additionally, provision of inflammatory milieu responsible for certain diseases was demonstrated to induce the cells to secrete regenerative factors. Intravenous infusion of human MSCs improved the cardiac function and decreased scarring in a mouse model of myocardial infarction. In the presence of a high level of inflammatory cytokines, human MSCs secreted excessive amounts of anti-inflammatory cytokine TNF-*α*-stimulated gene/protein 6 (TSG-6) that enhanced tissue regeneration [363]. Despite the fact that coculturing and direct cell-cell contact between MSCs and endothelial progenitor cells induced MSC differentiation toward a pericyte-like phenotype [44], it was demonstrated that intravenous administration of MSCs inhibited angiogenesis and endothelial cell proliferation, induced by cell-cell contact through modulation of the VE-Cadherin/*β*-catenin signaling pathways [364].

## 6. MSCs and Growth Factors

Growth factors are molecules that cause several biological effects, such as changes in motility, proliferation, morphogenesis, and survival of the cell [15, 365]. Various growth factors affect MSC properties (Table 3).

In mammals, three isotypes of TGF*β* are present: TGF*β*1, TGF*β*2, and TGF*β*3 [366]. The three isotypes are well-known inducer of MSC chondrogenesis that lead to proteoglycan and collagen type II deposition when applied as single factors [367, 368]. TGF*β* influences the proliferation and chondrogenic differentiation of MSCs [368–372]. TGF*β* plays a role through all phases of chondrogenesis, promoting mesenchymal condensation, chondrocyte proliferation, and ECM deposition and inhibiting terminal differentiation [373–375]. Moreover, TGF*β*1 has been reported to switch the human MSC fate from adipogenic to osteogenic when added under adipogenic culture differentiation conditions [376]. On the other hand, TGF*β*1 decreased the number of osteoprogenitor cells during the in vitro expansion of human BMSCs and downregulated ALP and STRO-1 expression [377]. These results suggest that TGF*β*1 effect could depend on the commitment state of the MSCs.

Bone morphogenetic proteins (BMPs) that belong to the TGF*β* superfamily play an essential role in regulating MSCs' proliferation and lineage-specific differentiation [378, 379]. BMP2, BMP4, BMP6, BMP7, and BMP9 induce osteoblastic differentiation of MSCs [380, 381]. MSCs exposed to these osteogenic BMPs increased the expression of ALP, osteocalcin as well as osteopontin, connective tissue growth factor, inhibitor of DNA binding, and Cbfa1/RUNX-2 [380–386]. BMP9 is considered as one of the most potent BMPs to induce MSC osteogenic differentiation [387–389]. Even though BMP2, BMP4, BMP6, BMP7, and BMP9 revealed the ability to induce adipogenic differentiation of MSCs [382], BMP2 [390], BMP4 [391], and BMP6 [392] promoted chondrogenesis only when applied in combination with TGF*β*. Although BMP3 stimulated MSC proliferation, it did not promote their adipogenic differentiation [393].

VEGF is known as a potent angiogenic factor that has been reported to increase prosurvival factors, phosphorylated-Akt, and Bcl-xL expression besides enhancing MSC proliferation in vitro [394]. Furthermore, VEGF favors MSC osteoblastic differentiation at the expense of adipogenic differentiation through regulating RUNX-2 and PPAR*γ*2 [395]. Additionally, in the presence of VEGF-A, MSCs differentiated into endothelial cells both in vitro and in vivo [396, 397]. Intracellular blockage of VEGF signaling by retroviral transduction of human MSCs to express a decoy soluble VEGF receptor-2 that sequesters endogenous VEGF in vivo resulted in spontaneous chondrogenic differentiation. Implanting transduced MSCs seeded on collagen sponges subcutaneously in nude mice activated TGF*β* signaling by blocking of angiogenesis and generation of a hypoxic environment that led to hyaline cartilage formation [398]. VEGF coinjection with BMSCs into a myocardial infarction heart mouse model led to increased cell engraftment and improvement of cardiac function as compared to injection of BMSCs or VEGF alone [394]. The proangiogenic effects of intramyocardial injection of FGF2 (bFGF) as well as intramyocardial and intravenous VEGF in a porcine model of chronic hibernating myocardium were further evaluated. The myocardial blood flow increased significantly only by the intramyocardial injection, which could be attributed to the diffusion of the factors from the point of injection and their ability to initiate the migration of cells [399]. FGF2 increased the migratory activity of MSCs through activation of the Akt/protein kinase B pathway [400]. These results were confirmed by analyzing the orientation of the cytoskeleton, where actin filaments acquired a parallelized pattern that was strongly correlated with the FGF2 gradient. Remarkably, FGF2 influence was confined not only to attracting MSCs but also in routing them as it has been revealed that low concentrations of FGF2 led to MSC attraction, while higher concentrations resulted in repulsion.

The FGF family includes members that affect MSC proliferation as well as differentiation where FGF2 and FGF4 increased proliferation potentials of BMSCs [401, 402]. FGF2 induced neuronal differentiation of human dental pulp MSCs [403] and stimulated chondrogenic differentiation of human MSCs [404, 405] and adipogenic differentiation of rat MSCs [406]. FGF2 promoted osteogenic differentiation of MSCs by inducing osteocalcin gene expression and enhancing calcium deposition [407, 408]. Additionally, a low dose of FGF2 enhanced the in vitro osteogenic differentiation of MSCs induced by BMP6 as well as bone formation in vivo [409]. However, there are contradictions in the literature about the exact role of FGF on MSCs. FGF2 was reported to inhibit mouse MSC differentiation by upregulation of Twist2 and Spry4 and the suppression of extracellular signal-regulated kinase 1/2 activation [410]. Moreover, FGF1 and FGF2 inhibited adipogenic and osteogenic differentiation of human BMSCs [411, 412]. FGF2 was also reported to inhibit osteogenic differentiation of mouse BMSCs at the early stage, promoted it in the medium phase, and maintained it in the later stage during osteogenic induction [413].

PDGFs are known to enhance cell proliferation and migration. There are four types of PDGFs (AA, BB, CC, and DD) [414]. PDGF-AA was reported to promote MSC migration and osteogenic differentiation [415]. PDGF-BB protected MSCs derived from immune thrombocytopenia patients against apoptosis and senescence, where PDGF-BB decreased p53 and p21 expression, while increasing the surviving markers' expression [416]. The combination of PDGF-BB, FGF-2, and TGF*β*1 led to synergistic enhancement of human MSC propagation with retained phenotypic, differentiation, and colony-forming unit potential [417].

Collectively, growth factors offer a promising approach to enhance MSC proliferation, differentiation, survival, and expansion. Choosing the proper growth factor is governed by three major criteria. First is the ability of the growth factor/s to prolong the proliferation in order to generate a sufficient number of MSC differentiation into the desired cell type. Second is the ability to replace the animal serum or xenographic substances. Finally is utilizing the properly localized and controlled method for delivering growth factors in vivo to take the benefit of their sustained release without inducing MSC uncontrolled proliferation and subsequent tumor formation [365]. Although using a single growth factor has advantages in increasing MSC proliferation, differentiation, and migration, combined growth factor treatment could provide more benefits due to possible synergistic effects on MSCs.

## 7. Risk of Tumorigenicity (Figure 2)

Every medical therapy carries some risk to the patients, and a careful weighing of the probable risks against the provided benefits should be carried out. Although the risk of tumorigenicity is far less with adult cells, with little evidence of tumor formation [418], it should never be neglected.

Stem/progenitor cells and cancer cells share some features suggesting a link between these two populations of cells, including long life spans, relative apoptotic resistance, and an ability to replicate for extended periods of time. Moreover, both share the same growth regulators and cell maintenance control mechanisms [419]. Stem/progenitor cell trafficking pathways seem to be further utilized by cancer cells for metastasis [420]. Stromal cell-derived factor- (SDF-) 1 impinges cancer cell behavior and migration and at the same time plays a role in stem/progenitor cell homing [421–423]. Also, CXCR2 and 4 receptors found on both stem/progenitor and cancer cells can influence both stem/progenitor cells' homing and cancer cells' invasion/metastases [419]. In addition, cancer and stem/progenitor cells have an inherent ability to evade host immune recognition [424]. Therefore, the malignant transformation of MSCs used in cell-based therapy might take place in the following circumstances: first during in vitro expansion of MSCs and second during genetic manipulation of MSCs.

### 7.1. *In Vitro* Malignant Transformation

In vitro expansion and culture of MSCs prior to cell administration may result in changes in their characteristics due to intracellular and extracellular influences. Harmful mutations during cell division as well as failure to correct these alterations may occur, causing tumorigenic transformation [425]. Some studies suggested that the tumorigenicity of MSCs increases proportionally with the length of in vitro culturing duration [426]. MSCs from different animals such as rat [427–429], rabbit [430], and cynomolgus [431] undergo spontaneous transformation during long-term in vitro culture. Moreover, these spontaneously transformed MSCs were found to be highly tumorigenic when inserted into immunodeficient mice [432, 433]. Spontaneous malignant transformation of mouse neural precursor cells was detected following ten in vitro passages, producing tumors in rodent brains [434].

An investigation studied the characteristics of the transformed MSCs (tMSCs) obtained from long-term culturing of rat MSCs. They revealed that tMSCs maintained typical MSC surface markers. Meanwhile, they exhibited a high proliferation rate with very limited senescence, lost contact inhibition property and mesodermal lineage potency, and subsequently acquired the ability for anchorage-independent growth [435]. The authors attributed the results to the increased levels of mutant p53 in the tMSCs that led to a significant upregulated expression of survivin, the main factor for the unlimited proliferation of transformed MSCs, and undetectable expression levels of the key senescence regulator p16 [435]. Moreover, silencing of the key regulator genes for cellular senescence such as p21 [436] and p16 [437], in addition to unscheduled epigenetic alterations, may be further key reasons for the cell to initiate this transformation [428].

Furthermore, long-term culture (exceeding five weeks) of human bone marrow- and liver-derived MSCs was evaluated for transformed cells. Four out of 46 batches had transformed cells that were able to induce sarcoma-like tumors in immunodeficient mice. High-resolution genome-wide DNA array and short tandem repeat profiling excluded the possibility of cell line contamination. Fortunately, the authors identified a gene expression signature using gene and microRNA expression arrays that may help to screen cultures for signs of early malignant transformation events. These genes include CKMT1A that was elevated over 10,000-fold and miR-182 and miR-378 that were upregulated nearly 500- and 100-fold, respectively, in transformed MSCs [438].

In contrast, several articles verified the absence of tumorigenic potential of cultured MSCs originating from different tissues even at advanced in vitro culture times [439–441]. Human MSCs from bone marrow, chorionic villi, and amniotic fluid were found to be nonprone to malignant transformation, following extensive in vitro expansion [442]. Moreover, human umbilical cord-MSCs were not susceptible to spontaneous malignant transformation during long-term in vitro culturing. Human umbilical cord-MSCs exhibited positive expression of human telomerase reverse transcriptase and did not exhibit shortening of the relative telomere length. Nevertheless, malignant transformation could still be prompted by chemical carcinogens as 3-MCA [443].

A systematic review that enrolled seven studies comprising 593 patients, 334 treated with MSCs and 259 as a control group without treatment, reported safe cell infusion with no oncogenesis in the follow-up period of 10 to 60 months [444]. Another systematic review reported no association between MSC implantation and tumor formation. The reported malignancies occurring in patients following implantation was related to ongoing or previous ones with no de novo formation [445]. On the other hand, evidence of tumor formation was noticed four years following fetal neural stem cell transplantation into the brain and the fluid surrounding it of a boy with ataxia-telangiectasia. By genetic typing, it was demonstrated that the tumor cells were of donor origin [446]. Similarly, eight-year postintraspinal olfactory mucosal cell autoimplantation for treating spinal cord injury, a young patient developed a spinal cord tumor mass autograft-derived. Seemingly, autologous treatment strategies could be more hazardous contradicting the expectation to be less immunogenic and more long-lasting than allogenic ones [447].

The in vitro potential tumorigenicity of MSCs could be related to genomic instability, accumulation of DNA damage, and loss of cell cycle regulation during long-term culture. The absence of transformation potential must be demonstrated before clinical use. Therefore, it is beneficial to decide during preclinical development whether the manufacturing process leads to chromosomal abnormalities using various assessment techniques for genetic stability [448].

### 7.2. MSC Malignant Transformation due to Genetic Modification

Genetic modification is the process of modifying or inserting new genetic materials (transgene) into specific cells to generate a therapeutic effect by correcting an existing abnormality or providing the cells with a new function into a cell [449].

MSCs can be genetically modified by viral and nonviral methods. These techniques have been proven to be a very significant advancement in treating various diseases such as neurological, blood, vascular, and musculoskeletal disorders and cancer (reviewed in [450]). Nonviral vectors comprise physical and chemical methods of gene transfer that can deliver more transgenes than viral methods and possess less stimulating effect on the immune system; however, their main drawback is the low transfection efficiency and transient gene expression [451, 452].

Viral vectors include retrovirus, adenovirus, adeno-associated virus, and lentivirus [453]. Viral vector genomes are modified by deleting some areas of their genomes so that their replication becomes deranged; therefore, they are considered to be safer. Yet, there are some limitations, including their confined transgenic capacity size and their marked immunogenicity that can prompt the inflammatory system causing degeneration of transduced tissue and insertional mutagenesis [454, 455]. “Insertional genotoxicity” is a key factor that should be considered when choosing a vector type and design for cell therapy. Insertions may cause dominant gain-of-function mutations (such as activation of protooncogenes flanking an insertion site) mediated by either enhancer and/or promoter elements in the vector or by aberrant splicing from the vector transcript. This is favored by the genetic structure of the retroviruses and the frequently used transfection agents. Since the experiments that monitored the insertional mutagenesis are often performed in rodents with relatively short life spans, the true mutagenic risk cannot be determined on the basis of vector choice and the total integration load in the transplanted cells alone; therefore, the true risk remains ill-defined. Primate animal models that are able to tolerate a larger number of transplanted MSCs and with longer life spans where transient transfections could be proposed [456] were used.

Yet, it should be clearly noted that any therapy involving genetic manipulations may result in MSC malignant transformation through either tumorigenic transformation of the transgene or disruption of the MSC's genome by the inserted transgenes, causing MSC subsequent transformation [457]. Stricter control and safety measures are required in the production of MSCs for cell-based therapy, taking into consideration that MSCs can further turn malignant as a result of long-term culture and due to genetic manipulation. Governing the cell handling procedures in order to minimize the risk of malignant transformation is ultimately needed. Unfortunately, studying cancer development is a long process and requires a long follow-up of treated patients to verify safety in this context.

## 8. Cell Delivery

### 8.1. Delivery Route

Among the challenges that hinder the clinical translation of stem/progenitor cell-based therapies is the uncertainty in the therapeutic efficacy of MSCs. This could be attributed to the paradoxical results obtained from both animal studies and clinical trials, showing controversial effectiveness, partly due to the method of MSC delivery [61, 458].

In general, cells could be introduced locally or systemically into the tissues. The optimal delivery method depends mainly on two factors, namely, whether the targeted disease is local or systemic [61] and the mechanism of action of the used cells [458]. Whether or not MSC optimal performance is achieved when present at the target site of injury/inflammation is hereby an important question. If MSCs should exert their function mainly through secretion of cytokines and growth factors in the circulation, i.e., having paracrine and autocrine effects or through regulating the local immune response [459–461], the presence of MSCs in the target site would not be necessary and systemic effects could be achieved using a cell reservoir [61]. But if the presence of MSCs at the target site is mandatory, for example, by differentiating into replacement cells [462], or through the local production of angiogenic or antiapoptotic factors [459], then the delivery route must place the cells at the target site or facilitate their migration to the site of interest [61].

Tissue defects are preferably treated with either local injection, for example, intramuscular, intramyocardial, or injection at the injury site of the spinal cord [463], or implantation of cell-loaded scaffolds [464]. Local cell delivery is currently the most promising in tissue-engineering applications, where cell-loaded scaffolds are locally transplanted at the target site. The use of 3D synthetic or natural scaffold biomaterials was shown to protect the cells from the aggressive in vivo environment, protect against substantial cell loss following systemic delivery due to the pulmonary “first-pass” effect, enhance MSC homing [61, 465], and promote functional integration and regeneration of the damaged tissues [466]. The local delivery of MSCs, applied in conjunction with or without biomaterials, has shown vast therapeutic efficacy in musculoskeletal regeneration for repair of osteochondrogenic diseases/disorders, including rheumatoid arthritis, osteoporosis, osteogenesis imperfecta, osteoarthritis, nonunion bone fracture, and craniosynostosis in preclinical and some clinical settings [464, 467]. In addition, direct surgical intramyocardial injection of MSCs and catheter-based transendocardial injection have been investigated in preclinical and clinical studies for the treatment of cardiovascular diseases, showing promising efficacy and safety [468–474], through allowing for higher cell retention rates and providing a targeted delivery route without requiring the availability of chemotactic factors [475]. Although local MSC delivery may be desirable and promising in specific applications, the need for certain procedures and/or surgery could be associated with some risks [476].

Systemic delivery of MSCs is adopted when the target disease is systemic or the need to regenerate several damaged tissues is present. Intravascular injection benefits form wider distribution of cells throughout the body and are being minimally invasive. Systemic delivery is divided based on the vascular route into intravenous (IV) or intra-arterial (IA).

The most commonly investigated method for delivering MSCs is the IV route [458]. Significant entrapment of the IV-delivered cells in the lungs results in a significant reduction in the numbers of cells reaching the organ of interest due to pulmonary “first-pass” effect [477, 478]. MSCs have an estimated diameter of 20–30 *μ*m [477, 479], and experiments with microspheres have demonstrated that most particles of this size are filtered out by the lungs [477]. Yet, the number of entrapped cells in the lungs could be decreased with the administration of a vasodilator [478, 479]. Aside from the effect of cell size, adhesion to the pulmonary vascular endothelium may also contribute to pulmonary cell trapping as was evident from IV delivery of MSCs in a rat model [477]. Thus, lung entrapment may explain the low engraftment of IV-delivered cells in clinical trials [458, 480].

IA delivery of MSCs in animals enhanced the engraftment of injected cells through bypassing the pulmonary entrapment [481–483]. In a rat model of transient ischemic stroke, IA injection of allogenic MSCs into the internal carotid artery showed the ability of the IA-transplanted cells to migrate into the ischemic brain, resulting in improved neurological function and reduction of the infarct volumes [484]. In addition, IA delivery of MSCs reduced the expression of calcineurin (CaN), a serine/threonine phosphatase which mediates neuronal homeostasis, after ischemic stroke in a rat model. CaN hyperactivation following ischemic stroke triggers apoptotic signaling. Thus, significant improvement in functional activity and normalized oxidative parameters were evident in rats receiving IA MSC treatment as compared to the stroke group [485]. Renal IA delivery of MSCs in a porcine renal ischemia-reperfusion model resulted in MSC distribution throughout the kidney, mostly in the renal cortex, particularly inside glomeruli, thus limiting off-target delivery. In addition, MSC viability in the kidney eight hours following IA infusion ranged between 70% and 80%, which could permit more efficient interaction with injured tissue and enhanced regenerative effect [486]. In a clinical trial investigating subjects with subacute spinal cord injury, IA delivery (via the vertebralis artery) of BMSCs resulted in greater functional improvement as compared to the IV route [487]. However, a careful balance between achieving high cellular engraftment without compromising blood flow due to arterial occlusion is mandatory [482].

In the treatment of cardiovascular diseases, intracoronary infusion of stem/progenitor cells is a relatively less complex technique. Still, the possibility of myocardial necrosis resulting from microvascular obstruction by the infused cells greatly questions the safety of this route [474]. A meta-analysis investigated the efficacy of four different routes of MSC delivery in acute myocardial infarction in swine and in clinical trials. The investigated routes of delivery included transendocardial injection, intramyocardial injection, IV infusion, and intracoronary infusion. Results showed the superiority of the transendocardial injection route due to both reduction in infarct size and improvement in left ventricular ejection fraction in preclinical and clinical trials [488].

Other routes of administrations are available for specific therapeutic applications [476]. Notably, the intranasal delivery route is proposed as an efficient and noninvasive route to the brain and for systemic administration to the central nervous system, demonstrating enhanced cellular retention and several improved neurological/psychiatric outcomes [489]. The intranasal delivery route depends on the ability of cells to bypass the cribriform plate through various routes, such as the olfactory bulb or the cerebrospinal fluid [490]. A study demonstrated that MSCs administrated via the intranasal route have the ability to migrate toward the injured cortex in a mouse model of traumatic brain injury. The authors employed superparamagnetic iron oxide tagged with a fluorescein isothiocyanate fluorophore as a noninvasive magnetic resonance imaging probe for MSC labeling and tracking [491]. In a rat model of Parkinson's disease, intranasal delivery of MSCs resulted in the appearance of cells in the olfactory bulb, cortex, hippocampus, striatum, cerebellum, brainstem, and spinal cord [492]. However, more experimental studies on the safety and efficacy of the intranasal delivery route of MSCs are needed; as to date, only few animal studies and no clinical studies are available.

A recently developed “cell spray” method [493] was investigated as a novel delivery method for transplantation of allogenic human ASCs in the porcine myocardial infarction model. This new cell delivery method was reported to be safe, feasible, and effective and resulted in successful transplantation of ASCs forming a graft-like gel film covering the infarct myocardium, significantly improving cardiac function [493].

### 8.2. Dose

Administration of an optimal cell dose is an important requirement to obtain therapeutic efficacy of MSCs' transplantation [466, 475, 494]. The determination of the pharmacologically optimally effective dose range is a critical issue for clinical translation of stem/progenitor cell-based therapies. Challenges in defining an optimal cell dose for specific therapeutic applications are related to the large variabilities in MSC clinical trials, including different disease categories, study design, target tissue/organs, types of MSCs used, manufacturing protocols, routes of delivery, and dosing employed [495, 496]. Thus, standardization of study design is mandatory to allow for better evaluation and correlation of results among similar clinical trials [496]. The variability in the optimal dosing identified by the various trials is primarily affected by the different routes of delivery employed. IV injection is the most commonly used and investigated method for delivering MSCs to the blood, being the least invasive method. As discussed earlier, due to entrapment of most injected MSCs in the lungs (pulmonary first-pass effect), IV has the highest average MSC dose, compared to cell doses employed with other routes of delivery. IA injection allows MSCs to bypass the pulmonary entrapment; thus, clinical trials employing this route have significantly lower average doses in a narrower range than IV. However, IA is used in a smaller number of trials as it is more invasive than IV. Consequently, local routes of delivery which locate the cells in a target site require lower average cell doses than the wider cell distribution in the body and faster wash-out following IV injection [497].

It is assumed that the number of administered cells should vary proportionally with the observed clinical efficacy. However, the data that had arisen from preclinical studies and clinical trials on the stem/progenitor cell dosage has yielded contradictory results [474, 475]. Recently, multiple dosing of stem/progenitor cells has been demonstrated to be more effective than the administration of a single large dose [498–500]. These studies show that the full benefits of stem/progenitor cell-based therapies could be underestimated or unnoticed if they are measured after a single dose. These results suggest that although the optimal cell dose remains indefinable, multiple dosing of stem/progenitor cells may provide therapeutic superiority in cardiac repair [475, 498]. This could be explained by the fact that a single large dose initially presents a high number of cells but soon gets “washed out,” while multiple dosing could offer a more durable cell persistence and paracrine signal for tissue repair, by replacing the cells that die after transplantation. Yet, repeated dosing using invasive delivery routes such as intramyocardial and intracoronary injections is considered unsafe. In such situation, a systemic route of delivery such as IV administration should be proposed [501].

### 8.3. Homing and Functional Integration

A major concern in systemic delivery of MSCs is that cells may become entrapped within organs that filter the blood (first-pass effect), for example, the liver, lungs, and spleen. To avoid this, several strategies to minimize lung entrapment (as discussed before) and to improve the homing of systemically introduced cells are employed [466]. Although there are numerous reports of stem/progenitor cells homing to injured tissue, the exact mechanism is not yet clear. Among the proposed factors were defective vascular architectures found in tumors [502] or leaky vasculature in injured tissues due to the effect of histamine and other inflammatory mediators [503], resulting in passive entrapment in the interstitial space; other biochemical and biomechanical factors could also be involved.

Homing of MSCs depends primarily on the chemokine receptor, C-X-C chemokine receptor type 4 (CXCR4), and its binding partner SDF-1, also known as C-X-C motif chemokine 12 (CXCL12) [481]. SDF-1 chemokine is released by the injured tissue and interacts with the chemokine receptors (CXCR4 and CXCR7) leading to the migration of MSCs to the injured tissue [481, 504]. Several other cytokines and growth factors, including IL-1 to IL-6, PDGF, VEGF, and BMP, are secreted by platelets, inflammatory cells, and macrophages arriving at the site of injury which could promote migration of MSCs [505]. Additionally, the released inflammatory cytokines TGF*β*1, IL-1*β*, and TNF-*α* in injured/inflamed tissue enhance migration by upregulation of matrix metalloproteases (MMPs) that cleave gelatin, laminin, and type IV collagen, constituting the basement membrane of blood vessels, promoting transendothelial migration of MSCs [481, 506].

Biomechanical factors could also contribute to MSC homing [507]. Intermittent hydrostatic pressure was shown to promote the migration of MSCs in vitro, which could be attributed to the increased concentration of SDF-1 released from MSCs in culture medium with increased hydrostatic pressure [507]. Mechanogrowth factor (MGF), an isoform of IGF-1, is further generated by cells in response to mechanical stimulation and plays a key role in regulating MSC function, including proliferation and migration [508]. Culturing of rat MSCs with MGF increased cell migration in a concentration-dependent manner by altering the mechanical properties of MSCs and activating the extracellular signal-regulated kinase (ERK) 1/2 signaling pathway in vitro. MGF-induced MSCs' migration increased the phosphorylation level of ERK 1/2, cell traction force, cell stiffness, and cell fluidization as compared with the control (without MGF). The activation of the ERK 1/2 signaling pathway and remodeling of the cytoskeletal structure to regulate rat MSC mechanics suggest the potential biomechanical and biological role of MGF in inducing MSC migration [508].

Migration of BMSCs is also affected by several chemical and mechanical factors [509]. Mechanical factors include hemodynamic forces applied to the walls of the blood vessel, in the forms of cyclic mechanical strain and blood shear stress, through focal adhesion kinase (FAK) and ERK 1/2 signals, SDF-1*α*/CXCR4, and c-Jun N-terminal kinase (JNK) and p38 mitogen-activated protein kinase (MAPK) pathways. Also, the elastic modulus (stiffness) of the ECM transmits complex biophysical signals that exert an important role in modulating MSC behavior, including promoting cell migration. The microgravity environment encountered during spaceflight was also shown to affect MSC migration, where simulated microgravity inhibited the migration of BMSCs via reorganizing or decreasing the expression of F-actin, increasing cell stiffness, and reducing SDF-1*α* [509].

Yet, any endogenous homing mechanism is insufficient, with less than 1% of delivered cells found in target tissues [458, 510]. Improving homing of the exogenous MSCs would greatly improve the functional integration of the cells into the target tissues [510]. Several different strategies for improving exogenous cell homing to the target site have been investigated (reviewed in [458, 510]). In brief, the different strategies can be divided into two main categories: (1) methods that increase the ability of stem/progenitor cells to respond to the chemotactic, homing, and migratory stimuli and (2) methods for modifying the target sites to enhance chemotaxis of stem/progenitor cells [510].

Stem/progenitor cell-based strategies include genetic modifications, priming of cells with growth factors and cytokines, cell preconditioning with hypoxia, treatment with certain chemical compounds that can trigger signaling pathways, and coating of the cell surface with double affinity antibodies or with homing ligands by streptavidin linkers and glycoengineering. Although different strategies have been introduced to increase the ability of stem/progenitor cells to respond to migratory stimuli, ex vivo expansion and manipulation may alter cell properties, such as proliferative capacity, differentiation potential, and genetic stability of cells, negatively affecting their safety at the clinical level. Thus, some prefer to modify the target sites, through designing more attractive environments to enhance stem cell recruitment. Target tissue-based strategies include direct transfection of target tissue with chemokine encoding genes, direct injection of chemokines or injection of ectopic chemokine expressing cells, the use of scaffolds as delivery vehicles, and application of electrical fields [510].

As discussed earlier, MSCs enhance tissue repair mainly through differentiating into replacement cells and/or paracrine effects [511], depending on therapeutic purposes of the transplanted stem/progenitor cells they could be introduced to act locally or systemically. For MSCs to achieve their intended therapeutic effect at the target site, functional engraftment of transplanted stem/progenitor cells is a prerequisite for achieving efficient regeneration via MSC differentiation to replace the damaged host cells. Even if cell therapy is used to provide paracrine factors or exosomes locally to support tissue repair or activate endogenous regeneration, initial engraftment of the transplanted cells to the target organ is necessary [512]. For the cells to integrate/engraft into the target tissue, cells need to adhere to the ECM of the tissues through the SDF-1/CXCR4 axis, failure of such interaction might trigger cell apoptosis in anchorage-dependent cells due to loss of contact with ECM, a process termed anoikis [504].

The limited functional integration of either autologous or allogenic stem/progenitor cell-based therapies remains a major clinical challenge. Following tissue injury, as in the case of myocardial infarction or cerebral stroke, the transplanted cells must replace billons of host dead cells to restore organ function, although the number of cells that actually home to and survive in the target organ is considerably low (as discussed before) [512]. In addition, cell survival into ischemic environment or inflamed tissue is quite low, due to lack of adequate oxygenation and the presence of inflammatory cytokine and ROS production after hypoxia and reoxygenation. Genetic engineering of MSCs with antiapoptotic and prosurvival factors such as the kinase Pim-1 was shown to enhance the repair of damaged myocardium in infarcted hearts [513].

Even in cases of physical engraftment of transplanted MSCs in injured tissues, successful functional integration, such as integration of transplanted cardiomyocytes with the host myocardium to allow a synchronized beating of the heart, is uncommon. Transplantation of more immature cells in a progenitor state might enable better in vivo functional integration [512]; however, transplantation of more immature stem cells caries the risk of tumorigenicity that could impair their therapeutic safety. That risk is lower if they are differentiated before transplantation, but differentiation results in increased immune recognition marker expression, triggering unwanted immune response. Thus, a balance between tumorigenicity and immunogenicity must be achieved [514].

Collectively, for more efficient and predictable outcomes of the transplanted MSCs, several important factors have to be taken in consideration. Choosing the optimal delivery route for each specific application, while carefully evaluating the merits and demerits of each delivery method, is recommended, based on the intended mechanism of action of the transplanted cells and the characteristics of the target organ/tissue. The recommendations of the optimal cell dose for each therapeutic application and delivery route are still not available, yet multiple dosing is suggested to offer enhanced and more predictable therapeutic effect through providing a prolonged cell persistence and paracrine signal for tissue repair. Adapting novel strategies to enhance the homing of exogenous MSCs are greatly needed to improve the functional integration of the cells into the target tissues, as any endogenous homing mechanism is insufficient for efficient integration of the transplanted cells. In addition, integrated personalized therapeutic approaches aimed at engineering the transplanted cells, to be more resistant to harsh environments and to enhance their survival and integration, might be necessary. Modification of the target site, for example, by rejuvenation of the vasculature and transplanting stem/progenitor cells together with bioactive factors and cytokines with/without biomaterials or mural cells, could aid in creation of a healthy paracrine environment, enhancing the functionality of transplanted cells [512].

## 9. Application of Biomaterials

As discussed above, the use of biomaterial-based 3D scaffolds for local delivery of MSCs could represent a promising and effective approach for modifying the target tissue and protecting the cells against the harsh environment in the diseased/injured tissues, enhancing cellular retention and functional integration. In addition, biomaterials can serve as carriers for bioactive molecules and growth factors that boost the regenerative capacity of MSCs such as VEGF, bFGF, HGF, IGF-1, and TGF*β* [465]. The optimal combinations of stem/progenitor cells and biomaterials that best suite each tissue and clinical therapeutic situation are still not clear. Significant efforts are being made to optimize compatible biomaterials with each stem/progenitor cell type for specific therapeutic applications [515].

In ischemic heart diseases, the natural architecture, vascularity, and metabolism of normal cardiac tissues are lost. Thus, cardiac tissue engineering, through engineering stem/progenitor cells on scaffolds, has been the ultimate purpose for cardiac repair [475, 516]. Hydrogels and/or bioactive agents are suggested to act as injectable delivery vehicles for MSCs to enhance the survival, retention, and efficacy of these cells in the injured myocardium. In addition, 3D patch-based systems are being widely investigated for myocardial repair to improve the therapeutic efficacy of stem/progenitor cell transplantation, while avoiding the risk associated with needle injection [465]. In a murine model of myocardial infarction, the application of BMSC-loaded poly(*ɛ*-caprolactone) (PCL)/gelatin cardiac patch supported the repair of the infarcted myocardium and enhanced the cardiac function. The MSC-loaded PCL/gelatin patch promoted the regeneration and angiogenesis of the injured myocardium, which may be attributed to the protection of the cells against the harsh hypoxic environment. In addition to the paracrine effect offered by the transplanted MSCs, the cytokines released enhanced the activation of the epicardium and recruited the endogenous c-kit^+^ cells [517]. A study [518] investigated the proangiogenic potential of cytokine-conjugated collagen patches seeded with human MSCs in a rat model of myocardial infarction. The investigated patches allowed prolonged cytokine release in the target site, together with enhancing cell infiltration and promoting functional neovessel formation, thus preserving cardiac function in the rat model.

There are many challenges facing the preparation of synthetic scaffolds that could mimic the natural cell microenvironment, which has directed the research interest toward utilizing naturally derived ECM itself, obtained through the process of decellularization [519]. Decellularized tissue scaffolds attract great interest in bone tissue engineering due to its natural 3D porous architecture and natural biochemical component arrangement, providing osteoinductive properties [520, 521]. However, the clinical translation of decellularized scaffolds is hindered by the challenge to balance between the optimal decellularization methods, to maintain the structural proteins that should have a positive impact on cell functions, while removing resident cells and genetic material that could cause an immunogenic response [519, 521].

Hence, the use of biomaterials could offer great benefits in enhancing the therapeutic outcomes, through supporting the cell integration and function aside from protecting them from the harsh in vivo environments of the injured/diseased tissues.

## 10. Effect of Antimicrobials, Local Anesthetics, and Other Drugs on MSC Properties

Different drugs and chemicals administrated, although being needed for specific therapeutic or prophylactic effects, could exert adverse effects or alter the properties of the transplanted MSCs, thus compromising/altering the effectiveness of MSC-based therapies.

### 10.1. Effect of Antimicrobials

The effect of several antimicrobial drugs, including antibiotics, antifungals, antivirals, antimalarials, natural peptides, and Chinese traditional drug extracts, on the differentiation potential of BMSCs has been reviewed in the literature [522]. Antibiotics or antimicrobials are commonly used to supplement culture media to avoid any bacterial contamination of the cell culture [523]. Isolation and cultivation of ASCs or oral MSCs usually involve the presence of the penicillin-streptomycin mixture [524]. Gentamycin is also commonly used. The use of amphotericin B is also suggested due to its widespread antifungal activity, but due to its cytotoxic effect on human cells, less toxic forms of the amphotericin B are currently available including a complex of amphotericin B with copper (II) ions (AmB-Cu2^+^) [525]. Unfortunately, antibiotics in a cell culture may change the regenerative potential and other biologic properties in many types of cells; for instance, penicillin-streptomycin mixture and gentamycin negatively affected the growth rate and target mRNA expression level of differentiating embryonic stem cells [526]. A study [527] investigated the effects of a penicillin-streptomycin mixture, amphotericin B, AmB-Cu^2+^, and their combinations on the proliferation and differentiation of ASCs in vitro. Data showed the effect of the investigated antibiotics on modulating the differentiation process, which is influenced by the duration of exposure and the combination of antibiotics employed [527].

Various antimicrobial drugs, although having a crucial role in the treatment of bone and joint infections and in prevention of postoperative infections, could exert specific effects on BMSC properties, specifically their differentiation potential. Cefazolin, a first-generation cephalosporin commonly used in arthroplasty to prevent infection, showed an irreversible negative effect on human BMSC migration and proliferation, in a time- and dose-dependent manner [528]. Rifampicin is a potent antibiotic commonly used in combination with ciprofloxacin in controlling orthopedic infections. High rifampicin concentrations, particularly higher than 16 mg/ml, exerted inhibitory effects on the in vitro proliferation and osteogenic differentiation of BMSCs [529].

### 10.2. Effect of Local Anesthetics

Intra-articular administration of amide-type local anesthetics is routinely performed during arthroscopic joint surgery to alleviate pain. In orthopedic cartilage repair operations, the delivery of human MSCs is often required via intra-articular injection, and it is common to introduce local anesthetics prior to, during, and following this procedure [530].

Lidocaine is one of the most commonly used amide-type local anesthetics due to its faster onset of action, superior safety profile, low cost, and wide availability compared to older local anesthetics. In vitro exposure of human ASCs to increasing concentrations of lidocaine resulted in a decreasing number of viable MSCs. Furthermore, reduction in cell proliferation was evident with the increasing exposure time, which suggests that lidocaine has a dose- and time-dependent cytotoxic effect on MSCs. MSCs subjected to lidocaine at various dilutions (2 mg/ml to 8 mg/ml) and exposure times (0.5 to 4 hours) showed upregulation of genes normally associated with responses to stress and cytoprotective mechanisms, while higher concentration of lidocaine (8 mg/ml and more) resulted in a significant drop in gene expression. Exposure of MSCs to high concentrations of lidocaine for prolonged periods was shown to negatively affect MSC viability, proliferation, and/or functions [531]. A recent study investigated the effect of lidocaine applied during tumescent local anesthesia prior to liposuction. Abdominal subcutaneous fat tissue was infiltrated with lidocaine-containing tumescent local anesthesia on the left and non-lidocaine-containing on the right side of the abdomen and harvested subsequently for cell analysis. Lidocaine showed no adverse effects on the distribution, cell number, and viability of ASCs [532].

Bupivacaine, ropivacaine, and mepivacaine are the members of the pipecoloxylidide group of amide local anesthetics, which differ in their onset of action, analgesic duration, and potency. Their analgesic potency increases in a ratio of 1 : 1.5 : 4 from mepivacaine to ropivacaine to bupivacaine, respectively [533]. Lidocaine, bupivacaine, ropivacaine, and mepivacaine were cytotoxic to rabbit ASCs during in vitro early chondrogenic differentiation, as evident by decreased viability and increased apoptotic rate of ASC monolayer cell culture experiments in a dose- and drug type-dependent manner. 1% lidocaine induced relatively lower cytotoxic effects on ASCs, and 2% mepivacaine and 1% lidocaine appeared to exhibit a less pronounced influence on chondrogenesis-associated mRNA expression [530].

In addition, local anesthetics could alter MSC secretory function, depending on the anesthetic dose and potency, along with the existing inflammatory environment [534]. A systematic review [535] evaluating the effect of various local anesthetics on different types of MSCs concluded that all amide-based local anesthetics exhibited cytotoxic effects on MSCs, and these effects were dependent on the dose, exposure time, and drug type. Cytotoxicity could also be cell type-dependent; however, there is currently insufficient evidence to support this hypothesis. Nevertheless, the study suggested that ropivacaine could offer less cytotoxicity than other types of local anesthetics and might be preferred for use in MSC-based therapy [535].

Future in vivo studies are crucial to better understand the interactions of these agents with MSCs in a more physiological environment, in terms of anesthetics' pharmacokinetics and the in vivo response and recovery of MSCs, to provide enough supporting evidence for future clinical trials [535].

### 10.3. Effect of Other Drugs

Heparin supplementation during culturing of human BMSCs was found to alter the cell biological properties, even at low doses, which warrants great caution regarding the application of heparin as a culture supplement for in vitro expansion of BMSCs. Also, heparin showed variable effects on gene expression and proliferation of human BMSCs in a donor-dependent manner, and MSCs harvested from patients receiving chronic heparin therapy could show altered properties [536].

MSCs have immunosuppressive properties (discussed above), and the presence of immunosuppressive drugs could offer synergistic effect, augmenting MSCs' immunosuppressive action [537, 538]. In vitro culturing of human BMSCs and ASCs in the presence of clinical doses of six widely used immunosuppressive drugs (cyclosporine A, mycophenolate mofetil, rapamycin, glucocorticoids, prednisone, and dexamethasone) was conducted to investigate their effect on immunosuppressive properties of MSCs. ASCs were less sensitive to the presence of immunosuppressive drugs than BMSCs. Glucocorticoids, especially dexamethasone, exerted the most prominent effects on both types of MSCs and suppressed the expression of the majority of the immunosuppressive factors tested [539].

Duloxetine (a serotonin and norepinephrine reuptake inhibitor) and fluoxetine (a selective serotonin reuptake inhibitor) are commonly used antidepressants for the management of major depressive disorders. Daily nontoxic concentration of both drugs exerted time-dependent effects on ASCs in vitro. In short-term exposure, both drugs influenced the proliferation and stemness properties of noncommitted ASCs, while following after 21 days of daily drug treatments, both cell proliferation and mesenchymal stromal cell marker expression were comparable to cells cultured in basal medium. Treatment with fluoxetine did not lead to morphological alterations during adipogenic or osteogenic differentiation of committed cells. Treatment with duloxetine resulted in slowing down lipid accumulation [540], which contradicts weight gain documented in patients treated for long durations [541] and increased mineral deposition, which could be correlated with the upregulation in gene expression of early and late osteogenic markers in ASCs treated with duloxetine [540].

Nonsteroidal anti-inflammatory drugs showed no interference with BMSC potential to proliferate and differentiate into osteogenic lineage in vitro, while inhibiting their chondrogenic potential [542].

In summary, it is evident that various drugs and chemicals used during MSC in vitro culturing and ex vivo expansion or during MSC transplantation could alter the cell viability, proliferation, properties, and/or function. The exact mechanism or consequences of each drug are still not clear, based on the currently available evidence in literature, and further future standardized in vitro studies, in vivo animal investigations, and clinical trials are greatly needed to carefully evaluate the effects of different drugs and chemicals used/needed during MSC-based therapies.

## 11. Conclusion

The results of MSCs' clinical applications are mixed and contradictory, preventing the advancement of MSCs into cell-based therapy. Although a considerable number of studies have proved the regenerative capacity of MSCs, significant limitations still exist hindering their usage as a clinically safe and efficient therapeutic approach.

Stem/progenitor cell-based therapy compromises variations related to the donor, their isolation, and expansion, as well as to the wide range of used media and their constituents and finally related to the recipients. All these variabilities suggest the need for developing a biological database, following reviewing the literature for the growth factors and cytokines associated with age, gender, health status, and immune response. Such biological map could enable the therapists to design a personalized protocol for each patient, considering the donor- and the recipient-related variations. Further, limiting and overcoming donor-related variations entail using standardized allogenic MSC transplantation following rigorous characterization and immunophenotyping [58, 61, 100]. Alternative cell sources as ASCs [57, 58], dental pulp MSCs [59], and stem/progenitor cell banking [61] should be considered for therapeutic use in aged patients instead of BMSCs.

Standardizing the materials used and the protocols utilized during fabrication is mandatory to alleviate the discrepancies during MSC fabrication. A chairside characterization facility should exist to examine the autogenous products from the patients (autoserum, for example) in order to overcome the immunogenicity and the time consumption associated with other alternatives. Moreover, the cell dose or cell delivery must be optimized according to the type and state of illness, utilized MSC predefined criteria, and condition of the patient. In addition, various drugs and chemicals used during MSC in vitro culturing and ex vivo expansion or during MSC transplantation could alter the cell viability, proliferation, properties, and/or function; thus, careful investigation of their effect on MSCs is mandatory.

**Figure 3:**
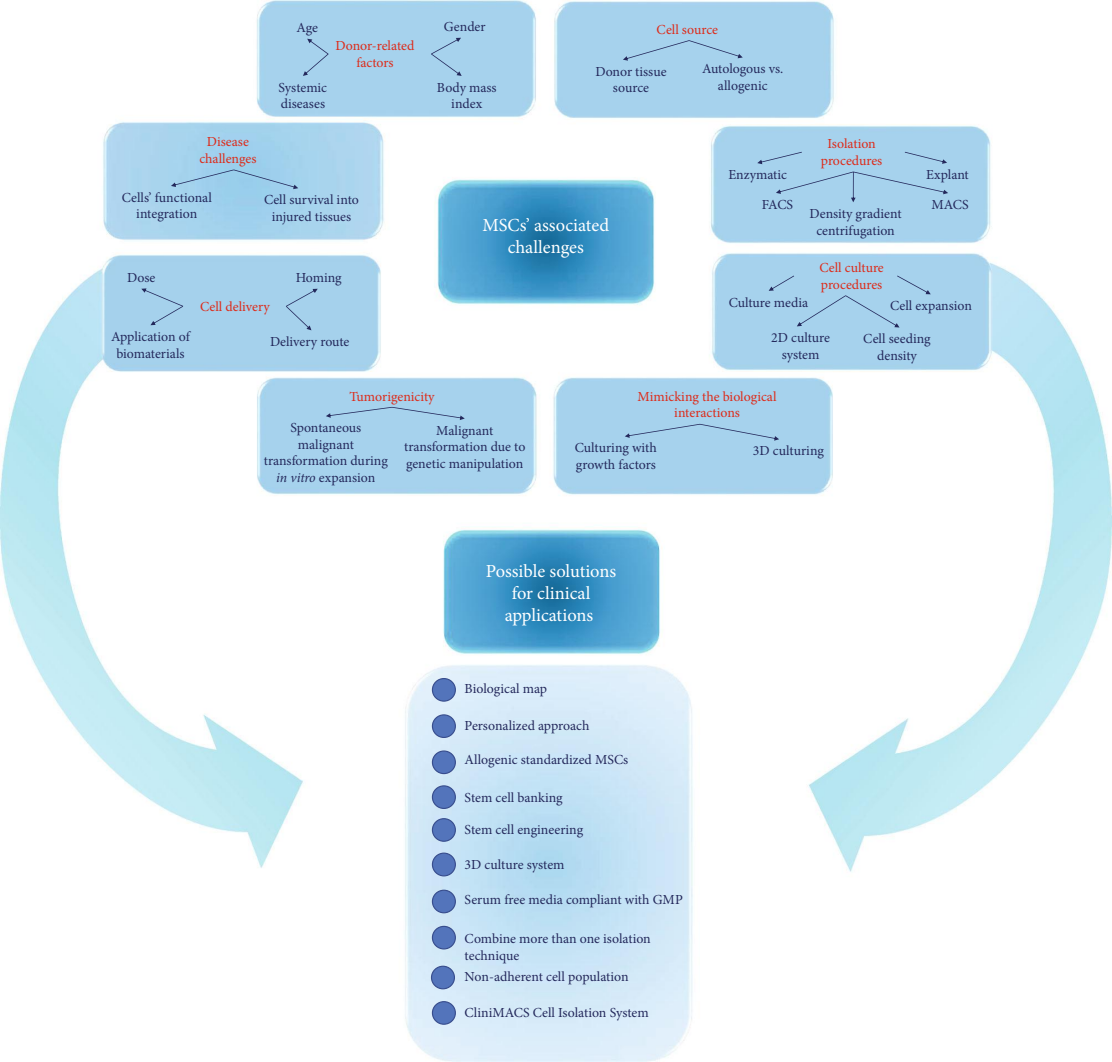
Challenges facing human clinical translation of MSC application and suggested strategies to overcome them.

Hence, for long-term therapeutic effectiveness and safety of MSC-based therapies, more research on both the preclinical and clinical levels has to be accomplished, focusing on optimizing the protocol for MSC isolation and in vitro expansion and preengineering to enhance their in vivo survival, differentiation, homing, and functional integration into the diseased/injured target site. In an attempt to prime the cells to be able to survive the harsh in vivo environment postinjury and to augment MSCs' biological and functional properties, preconditioning/pretreatment with hypoxia, growth factors, and/or drugs and genetic engineering of MSCs are an area of active research [543–547]. In addition, the establishment of personalized treatment approaches for patients adapted to their condition, disease state, and type of MSCs delivered is crucial. All these tactics (Figure 3) would greatly contribute to the successful and efficacious translation of MSC-based therapies into the clinical practice to be able to achieve the long-awaited regenerative and therapeutic role of MSCs.

## Figures and Tables

**Figure 2:**
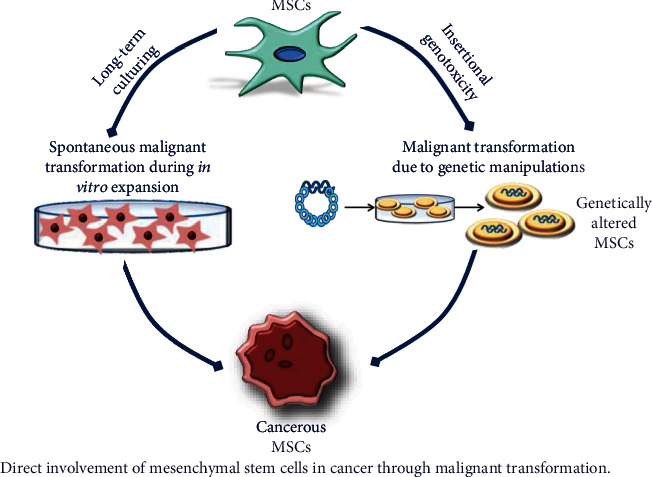
Direct involvement of mesenchymal stem cells in cancer through malignant transformation.

**Table 1:** Variability of MSC markers' expression with respect to source basis.

Mesenchymal stem cells		MSC source	Markers		Differentiation potential	Advantages	Disadvantages
			Positive	Negative			
BMSCs		Bone marrow aspirate [166]	CD29, CD44, CD73, CD90, CD105 CD166, Sca-1, and CD106 [548–551]	CD14, CD34, CD45, CD19, CD11b, CD31, and HLA-DR [548–551]	Adipocyte, astrocyte, cardiomyocytes, chondrocyte, hepatocyte, mesangial cells, myocytes, neuron, osteoblast, and stromal cell [552]	(i) Superior osteogenic and chondrogenic potential [142]. (ii) Multiple studies confirmed safety and efficacy of this type of cells [552].	(i) Bone marrow harvesting is invasive [143]. (ii) Fewer number of mesenchymal progenitors cells and lower proliferation rate as compared to MSCs from other sources [144, 145].

ADSCs		Adipose tissue obtained from liposuction [553]	CD29, CD44, CD13, CD166, CD73, CD90, CD105, CD34, CD49e, and CD10 [551, 554, 555]	CD31, CD45, CD14, CD11b, CD34, CD19, CD56, CD146, and HLA-DR [551, 554, 555]	Adipocytes, chondrocyte, myocytes,osteoblast, stromal cell [552]	(i) Less invasive isolation procedure [147]. (ii) Yield more progenitor cells [148, 149]. (iii) Have a higher proliferation rate as compared to BMSCs [145, 150].	(i) Adipogenic differentiation tendency [153, 154]. (ii) Low proangiogenic factors and cytokine secretion as compared to BMSCs [155, 156].

Dental stem cells		(i) Dental pulp stem cells isolated from dental pulp tissues of permanent teeth (ii) Pulp tissues of human shed deciduous teeth (SHED) (iii) Periodontal ligament stem cells isolated from periodontal tissues (iv) Dental follicle stem cells usually isolated from dental follicle surrounding third molar (v) Alveolar bone-derived stem cells, stem cells isolated from apical papilla at the apices of immature permanent teeth (vi) Tooth germ progenitor cells isolated from late bell stage third molar's tooth germs (vi) Gingival stem cells, isolated from gingival tissues [18].	CD105, CD73, and CD90 [164].	CD45, CD34, CD14, CD11b, CD79a, CD19, and HLA-DR [164]	Osteoblasts, chondroblasts, adipocytes, neuron, and angiogenic potential [161, 164]	(i) Relatively easier to isolate during routine dental treatments without teeth scarification [161] (ii) Possess higher proliferation rates, as compared to either BMSCs or ASCs [162, 163].	(i) Some types are inaccessible to isolate (ii) Difficult to isolate in sufficient amount (iii) Not readily feasible throughout the patient's life [165].

Perinatal tissues	Placenta MSCs	Human placenta [556, 557]	CD29, CD44, CD9, CD105, and CD166 [558]	CD34 and HLA-DR [558]	Hepatocytes, osteoblasts, adipocytes, insulin-producing cells, cardiomyocytes, and myoblasts [559, 560]	(i) Perinatal stem cells are acquired in a noninvasive manner. (ii) Perinatal stem cells are easily acquired from a material that was for long considered as medical waste (iii) They possess high proliferative rates (iv) Perinatal stem cells exhibit longer culture times, higher expansion, and delayed senescence and display high differentiation potential; additionally, placenta and umbilical cord provide a large number of progenitors as compared to MSCs from other sources [61, 166–170].	(i) Placental stem cell safety, contamination, tumorigenic transformation, and cellular changes following cell culture still merit further studying. (ii) Standardization of laboratory isolation protocols is still required [179].
	Umbilical cord MSCs	MSCs can be acquired from Wharton's jelly or umbilical cord blood [561].	CD29, CD44, CD73, CD90, CD105, CD106, CD117, CD133, and CD166 [562]	CD31, CD34, CD86, and HLA-DR [562]	Osteoblast, adipocytes, chondrocytes, hepatocytes, insulin-producing cells, cardiomyocytes, and neurons [563]		(i) Isolation and culture of stem cells from the umbilical cord are difficult (ii) Private banking is expensive and lacks strict regulations (iii) Lifelong storage is still unstudied [171].
	Umbilical cord blood MSCs	Cord blood is isolated prior to or immediately after delivery. It contains MSCs [561]	CD29, CD44, CD73, CD90, CD105, and CD166 [564, 565]	CD14, CD31, CD34, CD45, CD106, and HLA-DR [564, 565]	Osteoblast, adipocytes, chondrocytes, and myoblasts [566]		(i) Slow engraftment as compared to bone marrow [173]. (ii) Limited volume [176, 177] (iii) Low stem cell yield [178] (iv) Private banking is expensive and lacks strict regulations [172, 173]. (v) Lifelong storage is still unstudied [174, 175].

**Table 2:** Overview of main cell isolation/purification techniques used for MSC separation.

Isolation method	Isolation principle	Isolation technique
Enzymatic [11, 188]	Digestion of the tissue extracellular components by proteolytic enzymes	(1) Use of proteolytic enzymes such as collagenase and trypsin to digest the extracellular matrix. (2) After the extracellular matrix has dissolved, the released cells are seeded into culture dishes in growth medium.

Explant culture [11, 195]	Cell surface charge and adhesion to plastic surfaces	(1) The tissue is rinsed to remove blood cells. (2) The tissue is cut into smaller pieces of no more than a few millimeters in length. (3) The pieces are placed in culture dishes or flasks with growth medium. (4) Cells start to migrate out of tissue and adhere to the culture surface, and after several days, the tissue pieces can be removed.

Density-gradient centrifugation methods [567]	Cell size and/or density	(1) The sample is positioned on one or more layers having distinct densities, which are intermediate between those of the cells that are to be isolated and all other cells in the sample. (2) After that, centrifugation of the sample at the appropriate speed fractionates it into distinct phases between the different density layers.

Fluorescence-activated cell sorting (FACS) [181, 568]	Fluorescently labeled antibodies bind to surface or intracellular molecules	(1) Cells are labeled with a mixture of fluorescently conjugated antibodies. (2) The labeled cells pass aligned one by one through a nozzle which vibrates to produce droplets containing individual cells at a defined distance from the nozzle. (3) As the cells pass through the light source, a computer registers their individual light scatter and multiple fluorescent properties to detect cells that meet the preestablished criteria for selection. (4) A mild electrical charge is used to charge the drop where wanted cells are present. When the charged droplets pass between the two electrically charged metal plates, it deflects into a different collection tube.

Magnetic-activated cell sorting (MACS) [181, 567]	Magnetically labeled antibodies bind to surface molecules	(1) Cells are labeled with antibodies conjugated to biodegradable iron-based nanobeads. (2) The labeled cells pass through a strong magnetic field. (3) Cells conjugated with magnetic particles stay on the column, while nonconjugated cells pass though.

**Table 3:** Various growth factors and their effects on MSCs.

Growth factor family	Growth factor	Effect on MSC
TGF*β*	TGF*β*1	Increase proliferation & induce chondrogenic differentiation [369, 370].
	TGF*β*2	Induce chondrogenic differentiation [368, 372].
	TGF*β*3	Induce chondrogenic differentiation [371, 372].
	BMP2	Promote chondrogenesis [390], induce osteogenic differentiation [380–382], & induced adipogenic differentiation [382].
	BMP3	Stimulate proliferation [393].
	BMP4	Promote chondrogenesis [391], induce osteogenic differentiation [380–382], & induced adipogenic differentiation [382].
	BMP6	Promote chondrogenesis [392], induce osteogenic differentiation [380–382], & induced adipogenic differentiation [382].
	BMP7	Induce osteogenic differentiation [380–382] & induced adipogenic differentiation [382].
	BMP9	Induce osteogenic differentiation [380–382] & induced adipogenic differentiation [382].

VDGF	VDGF	Increase proliferation [394], favor osteogenic differentiation [395], differentiate into endothelial cells [396, 397], & induce chondrogenic differentiation [398].

FGF	FGF2	Increases migration [400], increases proliferation [401, 402], induces neuronal differentiation [403], and stimulates chondrogenic differentiation [404, 405], adipogenic differentiation [406], & osteogenic differentiation [407–409].
	FGF4	Increase proliferation [401, 402].

PDGF	PDGF-AA	Increases migration & osteogenic differentiation [415].
	PDGF-BB	Protect against apoptosis and senescence [416].
